# The foot of *Homo naledi*

**DOI:** 10.1038/ncomms9432

**Published:** 2015-10-06

**Authors:** W. E. H. Harcourt-Smith, Z. Throckmorton, K. A. Congdon, B. Zipfel, A. S. Deane, M. S. M. Drapeau, S. E. Churchill, L. R. Berger, J. M. DeSilva

**Affiliations:** 1 Department of Anthropology, Lehman College CUNY, 250 Bedford Park Boulevard W, Bronx, New York 10468, USA; 2 Division of Paleontology, American Museum of Natural History, CPW @ W. 79th Street, New York, New York 10024, USA; 3 Department of Anthropology, City University of New York Graduate Center, 365 5th Avenue, New York, New York 10016, USA; 4 Evolutionary Studies Institute and Centre for Excellence in Palaeosciences, University of the Witwatersrand, Private Bag 3, Wits, Johannesburg 2050, South Africa; 5 Department of Anatomy, DeBusk College of Osteopathic Medicine, Lincoln Memorial University, Harrogate, Tennessee 37724, USA; 6 Department of Biology, Southern Utah University, 351W Center Street, Cedar City, Utah 84720, USA; 7 Department of Anatomy and Neurobiology, University of Kentucky College of Medicine, MN 224 UK Medical Center, Lexington, Kemtucky 40536, USA; 8 Department of Anthropology, Université de Montréal, C.P. 6128, Succ. Centre-ville, Montréal, Quebec H3C 3J7, Canada; 9 Department of Evolutionary Anthropology, Duke University, 104 Biological Sciences Building, Box 90383, Durham, North Carolina 27708, USA; 10 Department of Anthropology, Dartmouth College, Hanover, New Hampshire 03775, USA; 11 Department of Anthropology, Boston University, 232 Bay State Road, Boston, Massachusetts 02215, USA

## Abstract

Modern humans are characterized by a highly specialized foot that reflects our obligate bipedalism. Our understanding of hominin foot evolution is, although, hindered by a paucity of well-associated remains. Here we describe the foot of *Homo naledi* from Dinaledi Chamber, South Africa, using 107 pedal elements, including one nearly-complete adult foot. The *H. naledi* foot is predominantly modern human-like in morphology and inferred function, with an adducted hallux, an elongated tarsus, and derived ankle and calcaneocuboid joints. In combination, these features indicate a foot well adapted for striding bipedalism. However, the *H. naledi* foot differs from modern humans in having more curved proximal pedal phalanges, and features suggestive of a reduced medial longitudinal arch. Within the context of primitive features found elsewhere in the skeleton, these findings suggest a unique locomotor repertoire for *H. naledi*, thus providing further evidence of locomotor diversity within both the hominin clade and the genus *Homo*.

## Abstract (web summary)

Hominin fossils reveal high diversity in the types of terrestrial bipedalism. Here, the authors show that the foot of *Homo naledi* from South Africa is predominantly human-like in morphology and inferred function and is well adapted for striding bipedalism.

## Graphical abstract

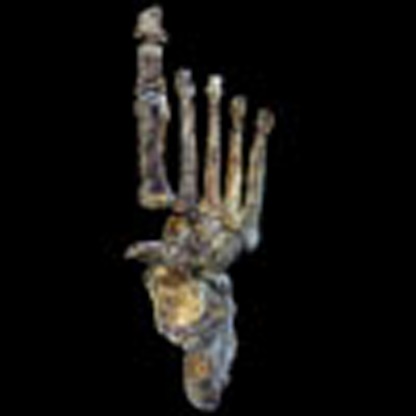

The *Homo sapiens* foot is highly adapted to striding bipedalism, and possesses a suite of anatomical features that functionally relate to this form of locomotion^1^,^2^. These include a non-opposable hallux, a medial longitudinal arch, a locking calcaneocuboid joint and an elongated tarsal region in conjunction with short, straight toes. In the last 20 years, our understanding of the evolution of human pedal function has become complicated by fossil discoveries that point to diversity in the types of terrestrial bipedalism found throughout the hominin clade, including several instances where contemporary hominin taxa possessed different combinations of pedal morphologies, indicating distinct differences in foot function^3^,^4^,^5^,^6^. In light of recent discoveries, there is also ambiguity concerning the locomotor affinities of basal members of the genus *Homo*^7^,^8^,^9^. It follows, then, that associated pedal remains of new fossil hominin taxa have the capacity to better our understanding of the complex evolutionary history of bipedalism.

Excavations at Dinaledi Chamber, located in the Rising Star Cave System, Gauteng, South Africa, have recovered 107 pedal remains that are assigned to the new hominin taxon *Homo naledi*^10^. These include a well-preserved right adult foot (Fig. 1), and isolated remains representing nearly all pedal elements (Supplementary Table 1). A subset of these elements can be provisionally assigned to two adult and two juvenile feet based on anatomical congruence and taphonomic association (Supplementary Table 2).

We show here that the foot of *H. naledi* is predominantly modern human-like in bony morphology and inferred function. When considered against the primitive features found elsewhere in the *H. naledi* postcranial skeleton^10^, these results indicate a locomotor repertoire that would have been distinct from that of other basal members of the genus *Homo*, such as *H. erectus* and *H. habilis*. The foot of *H. naledi* thus expands the range of locomotor diversity in both the hominin lineage and the genus *Homo*.

## Results

### Talus and calcaneus

Full descriptions of talus and calcaneus, as well as all other pedal elements currently assigned to *H. naledi*, are provided in Supplementary Note 1. The Dinaledi assemblage includes eight tali (Supplementary Fig. 1 and Supplementary Table 3) and four calcanei. The *H. naledi* talar trochlea is proximodistally flatter than that of *Australopithecus sediba*^5^, has only a moderate trochlear groove, and medial and lateral trochlear margins at a similar elevation to each other. The *H. naledi* trochlea is thus derived, as in *Au. afarensis* and later species of *Homo*, including *H. sapiens*^11^, and distinct from the markedly grooved, mediolaterally sloping trochlea of African apes, OH 8 (Supplementary Fig. 2) and several specimens from Koobi Fora^3^,^12^. In combination with the modern human-like wedging of the trochlea (Supplementary Fig. 3), these features suggest *H. naledi* had comparatively limited ankle mobility, and a modern human-like trajectory of the leg over the foot during the stance phase of gait^11^. Neither the talar head nor the proximal navicular facet is extended dorsally, indicating a modern human-like range of dorsiflexion and rotation at the talonavicular joint, which is limited compared with that of chimpanzees^12^,^13^. In addition, the average head and neck torsion angle of 38° is within the range of modern humans^14^, and implies a rigid midfoot with non-parallel calcaneocuboid and talonavicular joint axes during foot supination^15^. Relative to the trochlea, the neck and head of the tali are medially angled 20–26° (Supplementary Table 4), which is intermediate between average values for modern humans and extant apes^14^.

**Figure 1:**
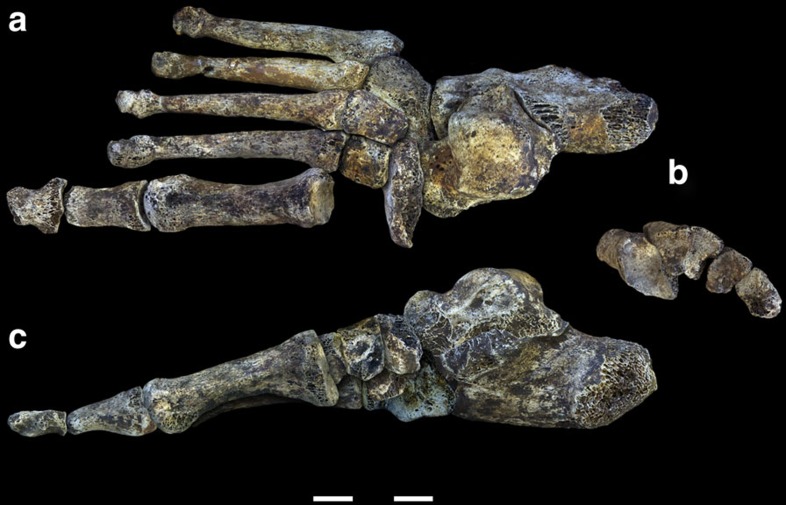
Digital reconstruction of the foot of *H. naledi*. All elements belong to Foot 1. (**a**) Dorsal view. (**b**) Distal view of the cuneiforms and cuboid showing transverse arch reconstruction. (**c**) Medial view showing the moderate longitudinal arch. Scale is in cm.

There are, however, several features of the *H. naledi* talus that are distinct from the modern human configuration. The lateral and medial malleolar facets are relatively flared (Supplementary Fig. 4), and, for the three intact adult Dinaledi tali, the angle of plantar declination of the talar head ranges between 10° and 18°, which is outside the human range of variation and within that of extant great apes (Fig. 1). Low head declination has been suggested to imply a low or absent medial longitudinal arch^14^, and has been linked to *pes planus* in a computed tomography-based study of modern human feet^16^.

Multivariate analysis of articular facet-based talar landmark configurations shows that the two most complete *H. naledi* tali (University of the Witwatersrand (UW) 101–148/149 & −1417) fall just at the edge of the human range of variation, and outside those of *Pan*, *Gorilla* and *Pongo* (Supplementary Fig. 5). Humans and *H. naledi* (along with *Au. afarensis* and the most intact talus from Koobi Fora, KNM-ER 1464) separate from the great apes (and OH 8) due to a flatter trochlea, less flared malleolar facets and a flatter posterior calcaneal facet^3^.

**Figure 2:**
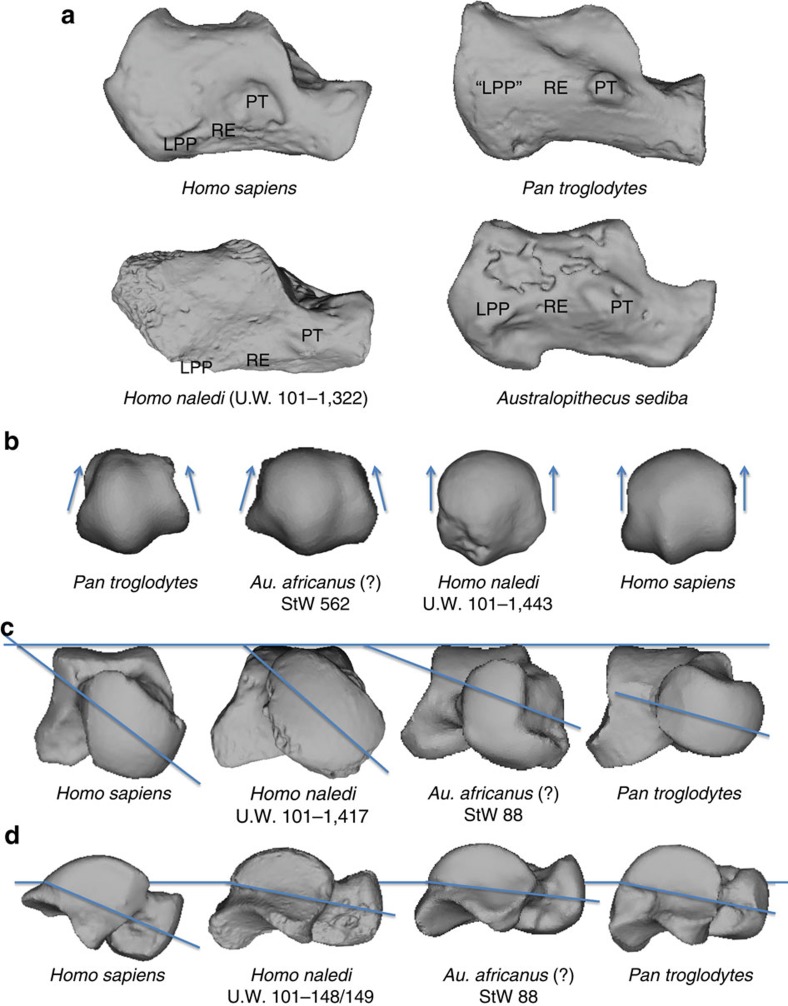
Salient features of the *H. naledi* foot. (**a**) The calcaneal anatomy of *H. naledi* resembles that of modern humans. The peroneal trochlea (PT) is markedly reduced and is connected to the lateral plantar process (LPP) by a diagonally oriented retrotrochlear eminence (RE), which helps position the LPP plantarly. In contrast, the calcaneus of chimpanzees and *Au. sediba* possesses a large and laterally projecting PT, with a more horizontally oriented RE and a dorsally positioned LPP. Calcanei have been scaled to roughly the same proximodistal length. (**b**) As found in modern humans, the *H. naledi* first metatarsal head is expanded dorsally, a product of parallel oriented lateral and medial rims of the articular surface. In contrast, the chimpanzee head tapers dorsally. StW 562 (*Au. africanus*?) is intermediate, with a mediolaterally wide metatarsal head, but still some dorsal tapering. Metatarsals have been scaled to roughly the same size. (**c**) The talus of *H. naledi* has human-like head and neck torsion relative to the talar trochlea. In contrast, the StW 88 (*Au. africanus*?) talus has low torsion, more similar to that found in modern apes. (**d**) Similar to that found in apes and in some australopiths (StW 88 shown here), the *H. naledi* head and neck are positioned dorsally relative to the trochlear body (line drawn passes through the proximal and distal extent of the articular surface of the talar trochlea). Modern humans tend to have a more plantarly oriented head and neck—an anatomy that has been linked to an arched foot^16^. Tali scaled to roughly the same size.

The most complete calcaneus of *H. naledi* (U.W. 101–1322) is well-preserved along much of its length, but exhibits some damage proximally such that only a small portion of the Achilles insertion on the tuberosity remains. The posterior subtalar joint is flat as in humans and *Au. afarensis*, and unlike that of *Au. sediba* and African apes, implying a limited range of motion^5^. The orientation of the sustentaculum tali is low compared with that of modern humans and the Omo calcaneus (33-74-896) (Fig. 2), and falls within the great ape range, along with the values for *Au. afarensis* and *Au. sediba*. This feature has been argued to relate to a low or absent medial longitudinal arch in non-human primates^1^. The peroneal trochlea is weakly developed, unlike in *P. troglodytes*, *Au. sediba*, *Au. afarensis*, *Au. africanus* and the Omo calcaneus, but is similar to that of modern humans and Neanderthals, perhaps indicative of reduced peroneal musculature^17^ in *H. naledi*. Although the lateral plantar process is not preserved, the retrotrochlear eminence suggests a modern human-like plantar position rather than the more dorsal one as in *Au. sediba* and chimpanzees^5^,^18^ (Fig. 1 and Supplementary Fig. 6). However, the calcaneal tuber is quite gracile, with a relative robusticity outside the range of modern humans (Supplementary Fig. 7 and Supplementary Table 5). Distally, the cuboid facet is dorsoplantarly concave, and has a proximally deep, medially placed depression for the cuboid beak. This modern human-like morphology is argued to help create a locking mechanism at the calcaneocuboid joint, yielding a rigid lateral column as the stance phase of gait approaches toe-off^19^.

### Cuboid, cuneiforms and navicular

Three cuboids, six naviculars, three medial cuneiforms, six intermediate cuneiforms and three lateral cuneiforms (Supplementary Tables 1 and  6–10) represent the midfoot morphology of *H. naledi*.

The cuboid is proximodistally elongated and exhibits a pronounced medioplantarly positioned beak, traits found in modern humans, *H. floresiensis* and the OH 8 foot^6^,^19^. UW 101–1418 presents a distinct Os peroneum facet on the proximolateral border of the peroneal groove, which may signify an oblique passage of the *M. fibularis longus* tendon and a stiffening of the midfoot^20^.

**Figure 3:**
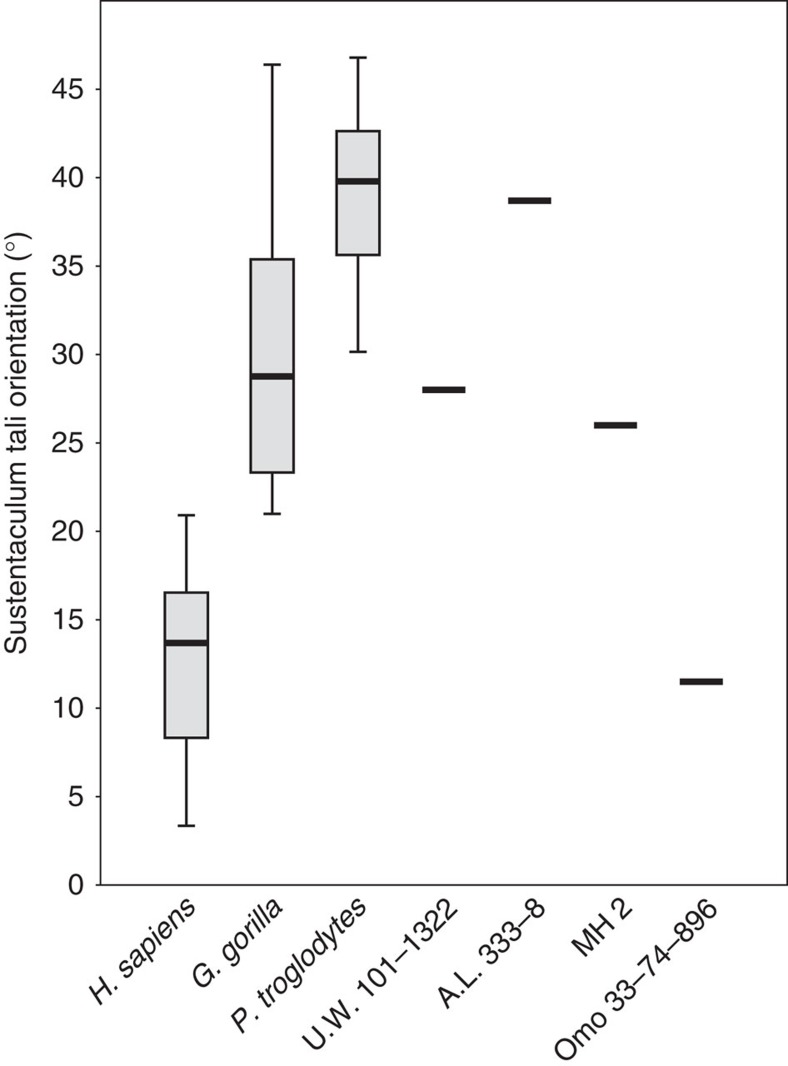
Orientation of the sustentaculum tali relative to the mediolateral axis of the calcaneal tuberosity. There is clear separation between African apes and *H. sapiens*. Dinaledi, along with *Au. afarensis* (A.L. 333-8) and *Au. sediba* (MH2) fall outside the human range of variation.

The only complete medial cuneiform (UW 101–1535) preserves a hallucial facet that, although slightly medially orientated, is relatively flat and in line with the tarsometatarsal row as in modern humans, OH 8 and StW 573 (Fig. 3), thus indicating an adducted hallux incapable of opposability. As in *H. sapiens*, the intermediate cuneiform facet is L-shaped. The Dinaledi intermediate and lateral cuneiforms are proximodistally elongated (Supplementary Fig. 8). On the lateral cuneiform, just distal to the cuboid facet is a small and slightly angled fourth metatarsal facet, indicating a modern human-like fourth metatarsal recession into the tarsal row^21^.

The naviculars are not well preserved, although the angulation between the cuneiform facets is modern human-like. The relative size of the medial tuberosity cannot be assessed.

### Metatarsals and phalanges

**Figure 4:**
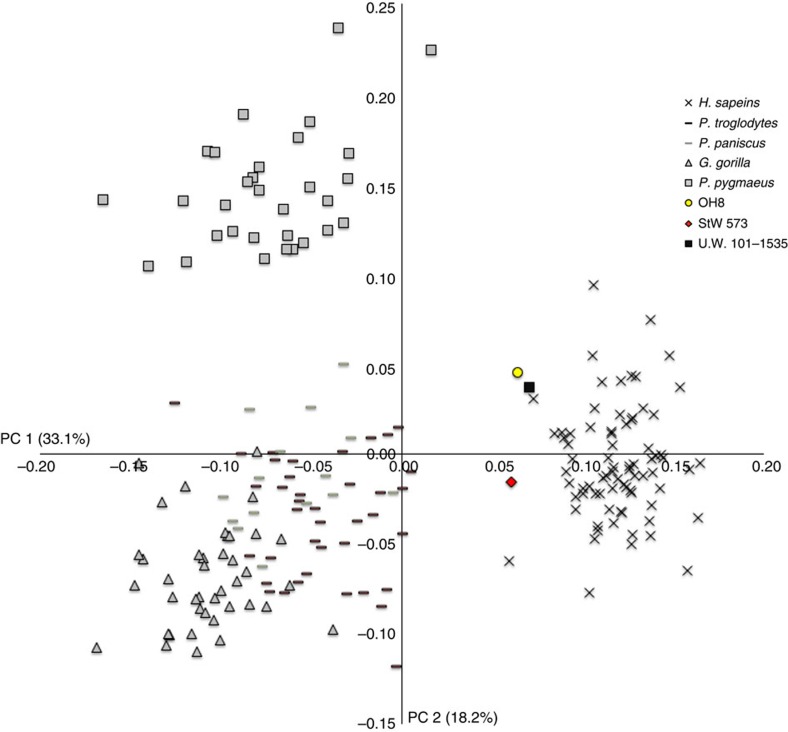
First two principal component scores of a generalized Procrustes analysis of medial cuneiform 3D landmark coordinates. Humans and the fossil hominins are separated from the great apes because of their flat, forward-facing hallucial facet. Dinaledi, along with OH 8 and StW 573 fall just within the *H. sapiens* range, and well outside that of the extant great apes.

**Figure 5:**
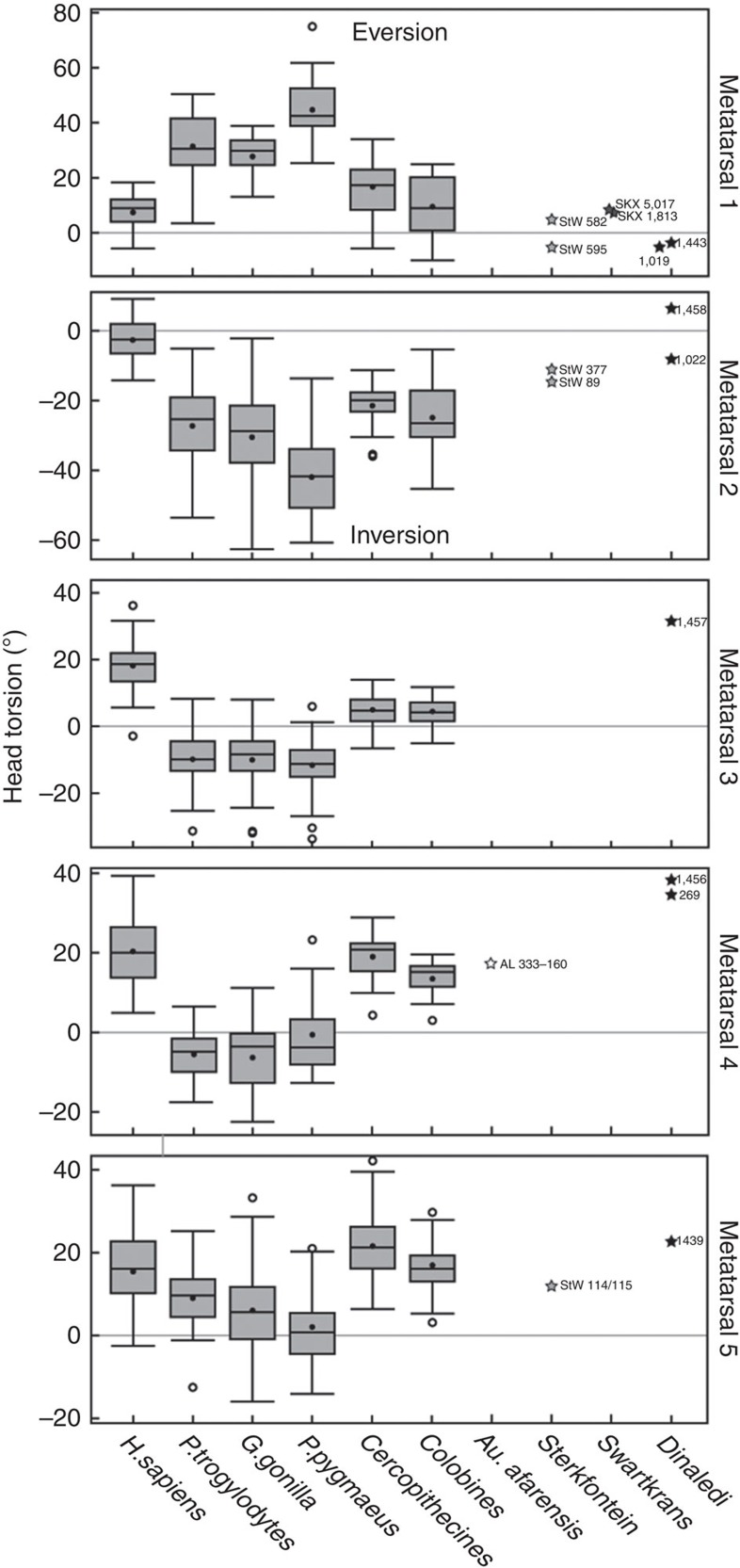
Torsion of metatarsals I–V for *H. naledi*. Darkened bars represent the median, boxes represent the 50% percentile limits and whiskers represent the highest and lowest values, excluding outliers. The values for the Dinaledi sample fall within the *H. sapiens* ranges of variation for all five metatarsals. Total sample sizes: *H. sapiens*: 50; *P. troglodytes*: 38; *G. gorilla*: 48; *P. pygmaeus*: 27; Colobines: 12; Cercopithecines: 25. *Note*: Sample sizes subtly vary per element according to the completeness of each skeletal specimen.

There are eight complete metatarsals, five of which were found in association as part of Foot 1 (UW 101–1443, 1456-8, 1439; Fig. 4 and Supplementary Tables 11–15), and a large number of metatarsal fragments. For Foot 1, the metatarsal robusticity ratio is 1>5>4>3>2, a condition shared with ∼60% of modern humans, but subtly distinct from the 1>5>3>4>2 configuration of OH 8 (ref. 22). Compared with the lateral metatarsals, the hallux is modern human-like in relative length (Supplementary Fig. 9), which is in contrast to the markedly short hallux of *H. floresiensis*^6^. The intermetatarsal facets are dorsally positioned and orientated dorsolaterally as in humans. The base of the 1st metatarsal is oriented perpendicularly to the long axis of the shaft and is more narrow mediolaterally than OH 8, StW 562 and 595 (but similar to *H. sapiens*). The diaphysial axes of the lateral metatarsals are oriented more proximolaterally to distomedially than OH 8, StW 89 and StW 114/115 (again, similar to *H. sapiens*). The bases of the 3rd metatarsals are gracile, whereas those of the 4th metatarsals are tall and robust (Supplementary Fig. 10). The dorsoplantarly flat articular surface of the cuboid facet of the 4th metatarsals (Supplementary Fig. 11) indicates that the lateral midfoot was modern human-like and rigid during heel lift, unlike the foot of apes and *Au. sediba*^23^. Metatarsal head torsion values generally fall within modern human ranges of variation (Fig. 5)^24^ and signify the presence of a transverse arch and at least an incipient medial longitudinal arch. The heads exhibit dorsal doming, and in many specimens there is a dorsal sulcus just proximal to the head, as in modern humans and australopiths, and indicative of increased loading in dorsiflexion^25^. The first metatarsal head is mediolaterally wide dorsally (Fig. 1), as found in modern humans^17^,^26^, but has a relative dorsoplantar (DP) height at the low end of the human range (Supplementary Fig. 12). The robust head of the first metatarsal is consistent with *H. naledi* having a modern human-like windlass mechanism during toe-off.

**Figure 6:**
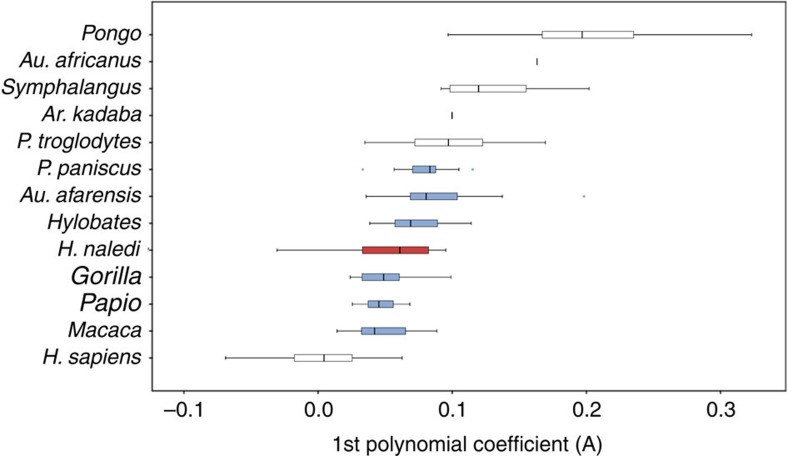
Longitudinal shaft curvature of pedal proximal phalanges. The Dinaledi sample (*n*=10) is shown in red. Differences between taxa were assessed using a one-way analysis of variance with a Bonferroni correction. Extant and fossil taxa that are not statistically distinct from that sample (*P*≤0.05) are shown in blue. Darkened bars represent the median, boxes represent the 50% percentile limits and whiskers represent the highest and lowest values, excluding outliers. Total sample sizes: *H. sapiens*: 85; *P. troglodytes*: 39; *P. paniscus*: 18; *Gorilla*: 17; *P. pygmaeus*: 40; *Hylobates*: 16; *Symphalangus*: 10; *Papio*: 10; *Macaca*: 21; *Ar. kadabba*: 1; *Au. afarensis*: 11; *Au. africanus*: 1.

There are 40 pedal phalanges in the *H. naledi* sample (Supplementary Tables 1 and 16–18). The proximal hallucial phalanx exhibits a robust base and narrow shaft similar to the adducted halluces of *Homo* and *Australopithecus*. The average dorsal canting angles of the proximal facets of the hallucial (*n*=2; 102.5°) and lateral proximal phalanges (*n*=4; 112.2°) also fall within the modern human range (Supplementary Table 19), indicating increased dorsiflexion at the metatarsophalangeal joints^25^,^27^,^28^. However, as with the *H. naledi* manual phalanges^29^, the proximal pedal phalanges exhibit significantly greater curvature than those of modern humans, falling within the range of *Gorilla*, *Hylobates*, *Papio*, *Macaca*, *Pan paniscus* and *Au. afarensis* (Fig. 6 and Supplementary Table 20). This anatomy may be indicative of elevated pedal grasping abilities in the *H. naledi* 2nd to 5th rays compared with modern humans.

## Discussion

The size of the Dinaledi pedal sample and the clear association among many of the pedal elements provides a rare opportunity to make inferences about foot function in a fossil hominin. The relatively complete Foot 1 has a power arm/load arm ratio of 40.8, which is close to the human mean and distinct from that of chimpanzees^30^ (Supplementary Table 21). Such a value indicates a foot capable of efficient weight transfer through to a terrestrial substrate. Given an ankle joint that is orthogonal to the long axis of the tibia^10^, a flat subtalar joint, diminutive peroneal trochlea, locking calcaneocuboid joint, limited dorsiflexion at the talonavicular joint, modern human-like metatarsal lengths, torsion and head proportions, and dorsiflexing phalanges, we infer the Dinaledi hominins' pedal function was broadly similar to that of modern humans. However, although the *H. naledi* foot was undoubtedly stiff during the stance phase of the walking cycle, the ape-like orientation of the sustentaculum tali and low talar head declination are features that may signal a relatively low medial longitudinal arch^1^,^14^,^16^, at least in Foot 1, although this hypothesis will require further testing of the relationship between arch height and bony anatomy of the foot. The relative flaring of the medial and lateral malleolar facets on the talus also point to increased stability when the foot was maximally inverted or everted. These characters, in combination with relatively low calcaneal robusticity and the more curved proximal pedal phalanges, result in a foot morphology that differed subtly from that found in modern humans.

This combination of primitive and derived features in the *H. naledi* foot (Table 1) is thus distinct when compared with the well-known foot assemblages OH 8 (refs 3, 12, 14, 22), StW 573 (refs 3, 31) and pedal remains assigned to *H. floresiensis*^6^, *Au. afarensis*^11^,^17^,^18^,^21^,^25^, *Au. africanus*^3^, *Au. sediba*^5^ and *Ar. ramidus*^20^. Given that there are no dates currently assigned to the Dinaledi sample, there are several possible scenarios when considering the *H. naledi* foot: (i) If the remains are from the early to mid Pliocene, they represent a foot more derived than in any other hominin taxon from that time period, indicating selection for human-like pedal function early in the hominin fossil record; (ii) a date from the late Pliocene/early Pleistocene would indicate a foot more derived than those of a similar age from Koobi Fora^3^, Olduvai^3^,^12^,^14^, Omo^32^ or South Africa^3^,^5^,^23^,^31^, and, although not fully modern human-like, perhaps most similar to that from Dmanisi^9^; (iii) a more recent date would indicate a foot that, although largely human-like, was distinct from that of *H. sapiens*, *H. floresiensis* and *H. neanderthalensis*, extending the range of variation in *Homo* foot morphology, and indicating possible stasis for primitive characters such as pedal phalangeal curvature.

Aside from that of *H. sapiens* and the Neanderthals, the Dinaledi foot possesses some of the most derived pedal morphologies in the hominin fossil record. Although there are members of the genus *Homo* known with primitive feet and relatively small brains (*H. floresiensis*^6^) and with derived feet and larger brains than *H. naledi* (for example, early *H. erectus*^9^), *H. naledi* is the first known hominin with this combination of such derived feet and legs with a small brain size^10^. Postcranially, the juxtaposition of such a derived foot in combination with the numerous primitive features found throughout the *H. naledi* skeleton is a unique mosaic previously unknown in the human fossil record. In particular, the primitive shoulder joint^10^ and curved manual phalanges^29^ indicate a decoupling of upper and lower limb function in *H. naledi* that offers an important insight into postcranial form and function that may have characterized basal *Homo*. Accordingly, the Dinaledi pedal remains provide further evidence of locomotor diversity within the hominin clade^3^,^4^,^5^,^6^, and expands that diversity within the genus *Homo*.

## Methods

### Descriptions

All Rising Star material was described by two or more individuals.

### Two-dimensional (2D) measurements

All linear measurements were taken with digital calipers; angular measurements from photographs or with a goniometer. Any measurement discrepancies in linear and angular measurements were rechecked by a third team member for accuracy. Unless otherwise specified, all proximodistal (PD) lengths were taken from the midpoint of the proximal articular surface to the midpoint of the distal articular surface, mediolateral (ML) lengths were taken from the midpoint of the medial surface to the medial point of the lateral surface, and DP lengths were taken from the midpoint of the dorsal surface to the midpoint of the plantar surface. The following are exceptions to this protocol: *lateral cuneiform PD* (measured by placing the caliper edge along the 3rd metatarsal facet and measuring to the proximolateral corner); *lateral cuneiform ML* (measured by placing calipers along the lateral edge and measuring to the distomedial corner); *talar neck PD* (measured parallel to the trochlea).

### Talar wedging

Talar wedging was measured as the ratio between the maximum ML width of the distal talar trochlea and the maximum ML width of the proximal talar trochlea. African apes were measured at the Cleveland Museum of Natural History (CMNH), American Museum of Natural History (AMNH) and Harvard Museum of Comparative Zoology (MCZ). Humans were measured at Kent State University (Libben) and the Hamann-Todd collection at the CMNH. Sample sizes are listed below the talar wedging graph. Original fossil tali were studied at the School of Anatomical Sciences and the Institute for Human Evolution (now Evolutionary Studies Institute), Johannesburg, Transvaal (now Ditsong) Museum in Pretoria, South Africa, Kenya National Museum (Nairobi). Casts of Ethiopian fossils were studied at the University of Michigan Anthropology Department.

### Talar angles

The horizontal angle of the head/neck, angle of torsion of the head/neck and angle of declination of the head/neck were compared with published measurements from Day and Wood^14^ (*Note:* Although Day and Wood^14^ call the plantar angulation of the talar neck and head an angle of ‘inclination', we prefer the term ‘declination' and use it throughout).

### Calcaneal robusticity

Calcaneal robusticity was measured as detailed in Latimer and Lovejoy^18^ and modified slightly in Zipfel *et al.*^5^. There is plantar damage to UW 101–1322 and the minimum area of the calcaneal tuber was estimated from a digital cross-section (using DeskArtes) of a surface scan of the original fossil. Tuber volume was then calculated as the product of the mimimum cross-sectional area of the tuber times the length of the tuber (from the midpoint of the proximal talar facet to the most proximal point of the calcaneal tuber). Calcaneal robusticity was calculated as the tuber volume divided by body mass. Body mass for the Foot 1 individual was calculated from regression-based equations of McHenry^33^ based on the talar trochlea width of the associated talus UW 101–1417. Comparative values were generated from data provided in Latimer and Lovejoy^18^ on modern apes and humans, with modified body masses in the apes from Smith and Jungers^34^. Data on calcaneal robusticity in *Au. afarensis* were from Latimer and Lovejoy^18^ and modified with a body mass generated from the regression-based equations of McHenry^33^ for talar trochlea width of A.L. 333-147—a not necessarily associated talus, but from a similarly sized individual as A.L. 333-8 and A.L. 333-55. Data on A.L. 333-147 are taken from Ward *et al.*^35^. Data for *Au. sediba* are taken from Zipfel *et al.*^5^.

### Tarsal elongation

Tarsal elongation was assessed as the maximum PD length of the bone divided by the maximum ML width of the bone in both the lateral cuneiform and the intermediate cuneiform. Chimpanzee, gorilla and orangutan measurements were obtained at the AMNH, MCZ and CMNH. Humans were from the Merida (Mexico) and Mistihalj (Montenegro) populations housed at the Harvard Peabody Museum of Archaeology and Ethnology (PMAE). Sample sizes are reported in the graphs themselves. Original fossils StW 573 (*Australopithecus* sp.) and UW 88–139 (*Au. sediba*) were measured at the School of Anatomical Sciences and the Institute for Human Evolution (now Evolutionary Studies Institute), Johannesburg, respectively. The OH 8 original fossil was studied at the Tanzania National Museum and House of Culture. A cast of the Hadar lateral cuneiform A.L. 333-79 was studied at the PMAE.

### Metatarsal proportions

Measures of the maximum PD lengths of *G. gorilla* (*n*=20), *P. troglodytes* (*n*=32), *H. sapiens* (*n*=97; all housed at the National Museum of Natural History, Washington DC), Metatarsal (MT) 1–3 lengths, were taken with digital calipers; measurements from LB1 were taken from the original specimen, and Skhul IV was taken from a cast. MT1/MT2 × 100 was found by dividing the length of MT1 by MT2 and multiplying the quotient by 100. MT1/MT3 × 100 was found by dividing the length of MT1 by MT3 and multiplying the quotient by 100. These data were plotted using PAST 3.0 (ref. 36).

### Metatarsal head dimensions

Relative metatarsal head dimensions were measured as described in the study by Latimer and Lovejoy^25^, in which the plantar cornua of the first metatarsal head did not factor into the PD height measurement. Because of ML erosion to the Dinaledi hominins, only the PD height of the first and second metatarsals were compared. Comparative data were generated from African ape specimens measured at the CMNH and humans measured at the PMAE. A cast of A.L. 333-115 was measured at the PMAE and results were comparable to those reported in the study by Latimer and Lovejoy^25^.

### DP curvature of MT4 base

DP curvature of the fourth metatarsal base was measured as described in the study by DeSilva^37^. Briefly, the base of the fourth metatarsal was depressed into a carpenter's contour tool in the coronal plane. Fossils were digitally sectioned using DeskArtes 3Data Expert 9.1 from high-resolution surface scans of the original fossils taken with a Next Engine desktop scanner. The maximum depth of the curvature was divided by the maximum DP height of the base: flatter bases resulted in a lower value; more convex bases a higher value. African apes and humans were both measured at the Cleveland Museum of Natural History (sample sizes reported in the graph itself). Data for A.L. 333-160 are from Ward *et al.*^21^, and UW 88-22 from DeSilva *et al.*^23^. StW 485 and OH 8 values are from DeSilva^37^.

### Metatarsal torsion

The methods used are described in Drapeau and Harmon^23^. Surface laser scans were made of the complete metatarsals, using ScanStudio software and a NextEngine laser scanner (NextEngine Inc.). Scans were imported into Geomagic, where four landmarks were placed, two delineating the major axis of the metatarsal head and two delineating the major axis of the base. The angle created by these two lines in the coronal plane represents the lateral torsion of the metatarsal. Torsion and intermetatarsal articular facet orientation were used to align the metatarsals in virtual space and model the shape of the transverse arch. Comparative specimens were measures at the Cleveland Museum of Natural History, National Museum of Natural History, Washington, DC, Anthropological Institute of the University of Zurich and the Canadian Museum of Civilization, Gatineau, QC, Canada. All measurements on fossils were taken from the original specimens, except for the Dinaledi specimens, which were taken from laser surface scans.

### Metatarsal robusticity

This has been carried out using the method of Archibald^21^ in which the metatarsal robusticity index is defined as [(mid-shaft diameter × 100)/length]. The mid-shaft diameter was calculated as mid-shaft circumference/π. The metatarsal dimensions used are defined as follows:

*Metatarsal 1*. Length is measured from the most distal point on the upper part of the proximal articular surface to the most distal point on the distal articular surface. Circumference of the mid-shaft is measured at a point mid-way between the most distal point on the proximal articular surface to the most distal point on the distal articular surface.

*Metatarsals 2–4*. Length is measured from the most dorsolateral point on the posterior articular surface to the most distal point on the distal articular surface. Circumference of the mid-shaft is measured at a point mid-way between the most dorsomedial point of the proximal articular surface to the most distal point of the distal articular surface.

*Metatarsal 5*. Length is measured from the most lateral point on the proximal articular surface to the most distal point of the distal articular surface. Circumference of the mid-shaft is measured at a point mid-way between the most medial point on the proximal articular surface to the most distal point of the distal articular surface.

### Phalangeal curvature

All phalangeal curvatures were quantified using high-resolution polynomial curve fitting (HR-PCF) methods^38^. Unlike traditional curvature quantification techniques (that is, included angle, normalized curvature moment arm) that model curvature as an imaginary circular line passing through the center of a bone, HR-PCF models the surface curvature of the bone and can fit a polynomial function to either the dorsal or ventral surface of a phalanx. In the case of the Dinaledi pedal phalanges, the ventral surfaces of many phalanges were interrupted by flexor sheath ridges that create irregularities in the outline of shaft curvature, so the more regular dorsal margin of the outline was chosen for polynomial fitting. Although it could be argued that the dorsal and ventral curvatures are responses to different loading regimes, they are highly interdependent and associated with the same positional behaviour; the dorsal curvature is also simpler. Elements were photographed in a lateral and standardized orientation. JASC PSP (Corel Corporation, 1600 Carling Avenue, Ottawa, Ontario K1Z 8R7, Canada) image editing software was used to convert the resulting 2D images into simple digitized outlines. These digitized outlines contain thousands of individual pixels, each having its own, paired coordinates. End points were selected for each dorsal contour to represent the limits of a discrete 2nd order curve and the co-ordinates of the individual pixels comprising the selected portion of the dorsal contour were used as data points to generate a best-fit 2nd order polynomial function with three coefficients defined as *y*=*Ax*^2^+*Bx*+*C*. The three resulting coefficients (*A*,*B*,*C*) can be used as the raw data in a statistical analysis. The first coefficient (*A*) expresses the nature and degree of the longitudinal curvature, whereas the second (*B*) and third (*C*) reflect aspects of the orientation of that curve with respect to the rest of the element (that is, element rotation, element position in 2D space). Given the limitations of coefficients *B* and *C* to represent meaningful information about the magnitude of phalangeal shaft curvature, only the 1st (*A*) polynomial coefficient was considered in statistical analyses performed in the present study.

Although any order of polynomial can be used with HR-PCF methods, a second-order polynomial was chosen over a higher-order polynomial functions because second-order curves (for example, longitudinal phalangeal shaft curvature) have no structural points of inflection, unlike third-order curves and above, which impose either one or more points of inflection. The coefficients of higher-order polynomials (that is, 3rd–6th order) are very sensitive to whatever irregularities exist in the contours of anatomical curves.

### Three-dimensional (3D) measurements

*x,y,z* homologous landmark configurations for the talus and medial cuneiform were collected according to protocols defined in Harcourt-Smith^39^. All landmarks were collected with a Microscribe digitizer by W.E.H.H-S. Coordinate date was superimposed using generalized Procrustes alignment, which controls for translational and rotational differences and adjusts for size. Aligned coordinates were subjected to a principal components analysis. All generalized Procrustes alignments and principal components analyses were done in *morphologika* 2.5 (ref. 40). Angulation between articular facets and other anatomical structures on the talus and calcaneus were collected using laser surface scans of the fossils and the software Geomagic Control 2014 (Geomagic Solutions), which allows a best-fit plane to be fitted to a selected area, or an axis to be determined using two landmark points.

All non-human extant samples for the 3D analyses are wild shot adults housed at AMNH, CMNH, MCZ, National Museum of Natural History (Smithsonian), Natural History Museum (London), Powell-Cotton Museum (Kent, UK) and the Royal African Museum (Tervuren, Belgium). Human samples were collected at the AMNH and the Dart Collection, University of the Witwatersrand. Original fossils were studied at the School of Anatomical Sciences and the Institute for Human Evolution (now Evolutionary Studies Institute), Johannesburg, the Transvaal (now Ditsong) Museum (Pretoria, South Africa) and the Kenya National Museum (Nairobi). Primary casts of Ethiopian fossils were studied at the Institute of Human Origins (Arizona State University) and at the Musée de l'Homme, Paris. Primary casts of Tanzanian fossils were measured at the Natural History Museum, London. Sample sizes for the talus 3D coordinates are: *H. sapiens*=89; *P. troglodytes*=44; *P. paniscus*=15; *G. gorilla*=42; *P. pygmaeus*=43. For the medial cuneiform 3D coordinates, they are: *H. sapiens*=77; P*. troglodytes*=40; *P. paniscus*=15; *G. gorilla*=41; *P. pygmaeus*=32. For the malleolar facet angle, they are: *H. sapiens*=26; *P. troglodytes*=25; *G. gorilla*=18. For the sustentaculum tali angle, they are: *H. sapiens*= 26; *P. troglodytes*=25; *G. gorilla*=23.

## Additional information

**How to cite this article:** Harcourt-Smith, W. E. H. *et al.* The foot of *Homo naledi*. *Nat. Commun.* 6:8432 doi: 10.1038/ncomms9432 (2015).

## Supplementary Material

Supplementary Information Supplementary Figures 1-12, Supplementary Tables 1-21, Supplementary Note 1 and Supplementary References

## Figures and Tables

**Table 1:** The mosaic foot of *Homo naledi*.

**Modern human-like anatomy**	**Intermediate anatomy**	**Ape-like anatomy**
Power arm/load arm ratio	Talar neck horizontal angle	Talar head/neck declination
Talar wedging	Flaring of talar malleolar facets	Sustentaculum tali orientation
Talar head and neck torsion	Calcaneal robusticity	
Talar trochlea margins even	Metatarsal 3 base height	`
Low talonavicular range of motion	Proximal phalangeal curvature	
Small peroneal trochlea		
Lateral plantar process position		
Flat subtalar joint		
Locking calcaneocuboid joint		
Int. and lat. cuneiform elongation		
Adducted hallux		
Int. and med. cuneiform articulation L-shaped		
4th Metatarsal base DP flat		
Metatarsal length proportions		
Metatarsal head proportions		
Metatarsal torsion		
Metatarsal 4 base robusticity		
MT 1 head dorsally expanded		
Dorsally canted phalanges		

DP, dorsoplantar; Int., intermediate; lat., lateral; med., medial.
