# Supplementary material for: The foot of Homo naledi
Source: Nat Commun. 2015 Oct 6;6:8432. doi: 10.1038/ncomms9432 (PMC4600720; doi:10.1038/ncomms9432)
Supplement: Supplementary Information — Supplementary Figures 1-12, Supplementary Tables 1-21, Supplementary Note 1 and Supplementary References [file ncomms9432-s1.pdf]

Supplementary Figures

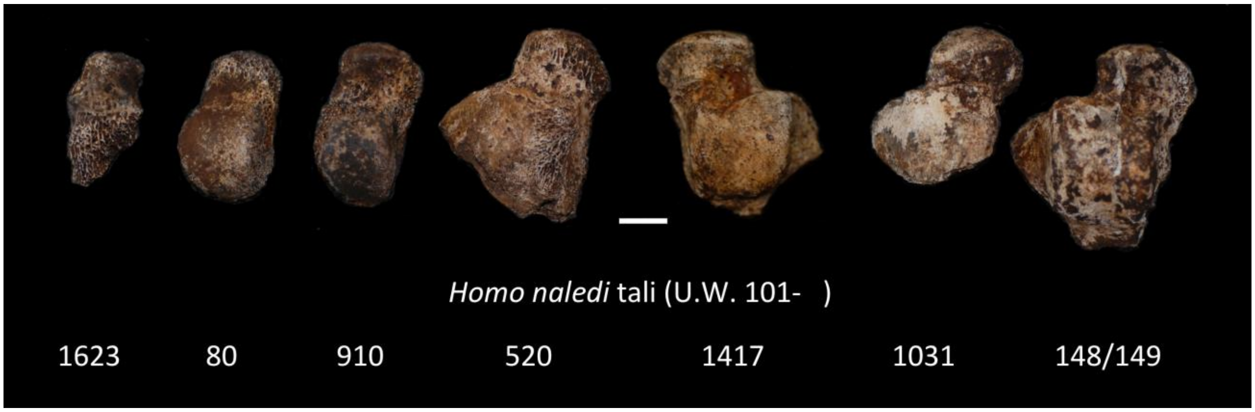

Supplementary Figure 1 | Tali from Dinaledi Chamber. Seven tali from *Homo naledi* are shown in dorsal view and arranged from smallest (to left) to largest (to right). U.W. 101-1623, U.W. 101-80, and U.W. 101-910 are from immature individuals, whereas the others are from adults. Scale bar is 1cm. Talus fragment U.W. 101-1215 is not shown here.

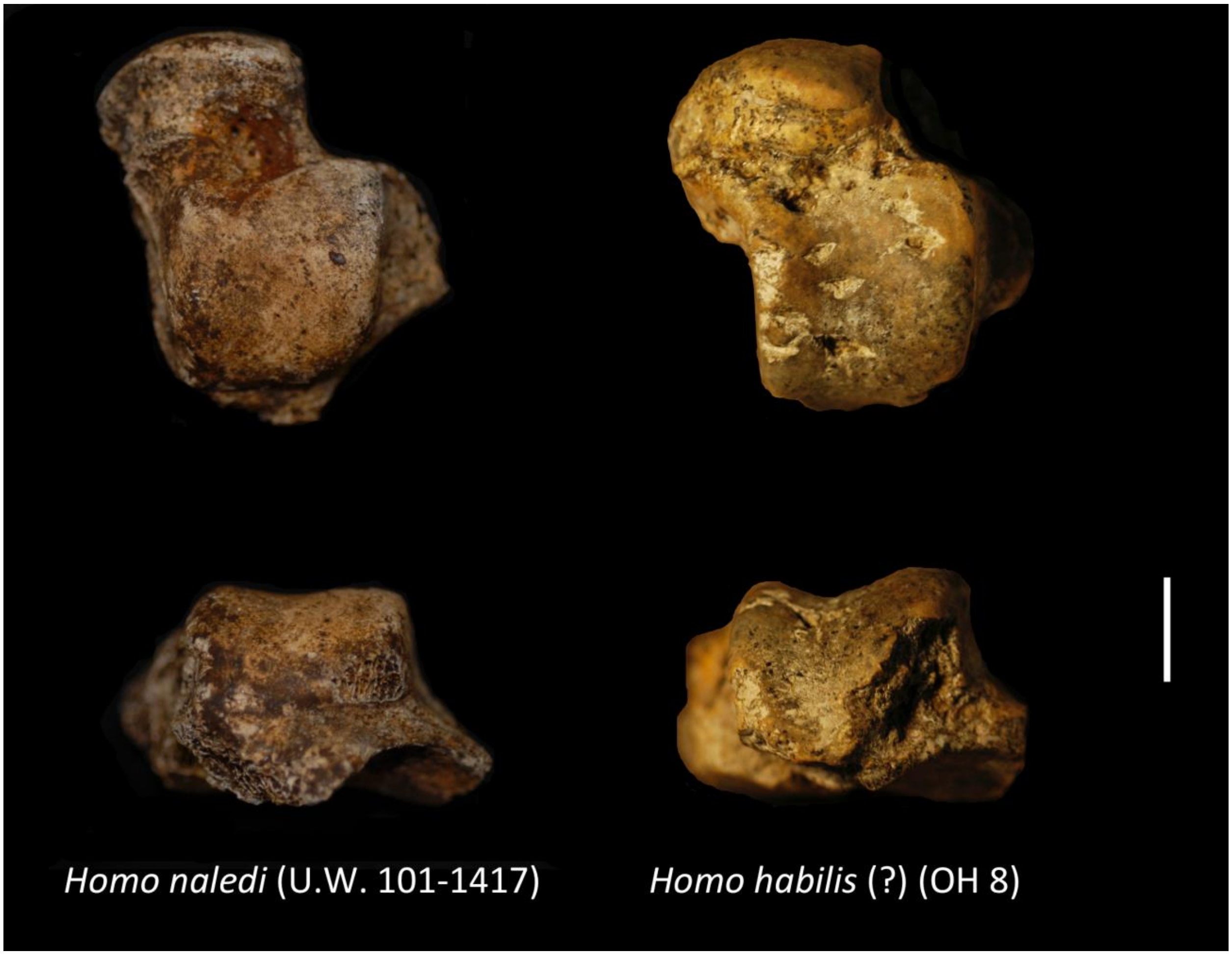

Supplementary Figure 2 | U.W. 101-1417 talus compared to OH 8. The talus of *H. naledi* is compared to OH 8 (often referred to as *H. habilis*) in dorsal (top) and proximal (bottom) views. Notice that compared to U.W. 101-1417, the OH 8 talar head and distal trochlea twists medially. Additionally, in proximal view, the differently shaped trochlear surfaces are apparent. *H. naledi* possesses a mediolaterally flat surface with only a weak midline groove, whereas OH 8 possesses a deeply keeled trochlear surface and an elevated lateral rim. Scale bar 1cm.

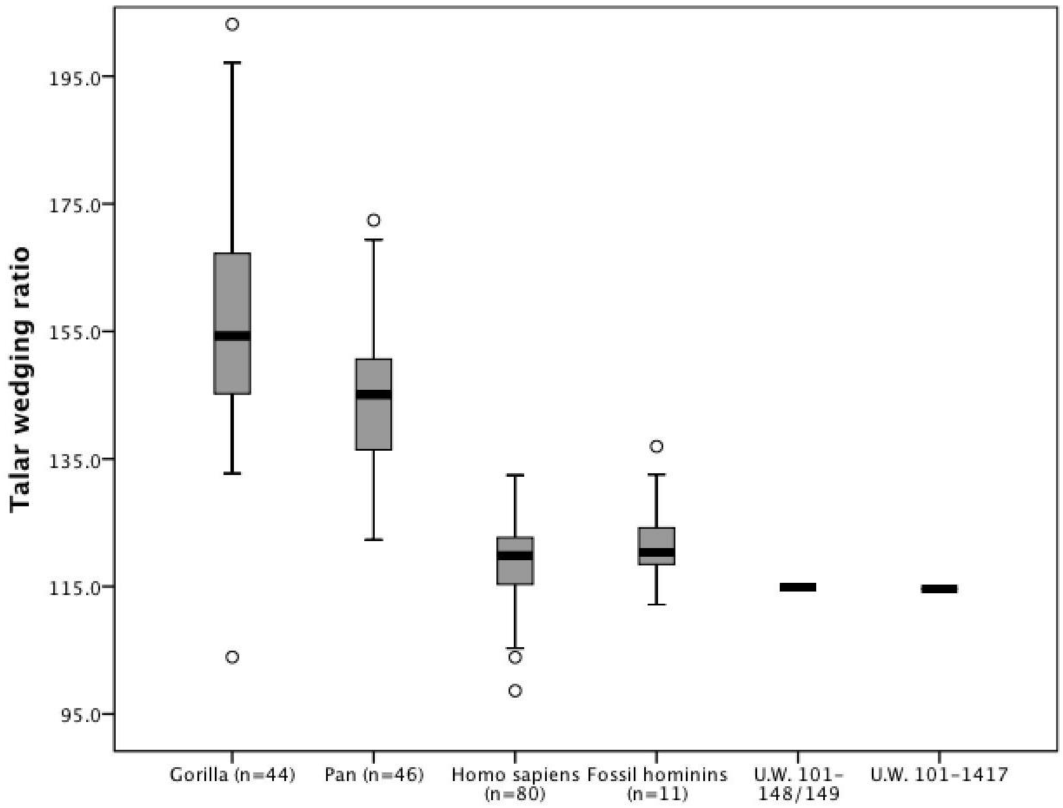

Supplementary Figure 3 | Talar wedging. African apes have a strongly wedged talus, which may help dissipate forces during flexed ankle vertical climbing[1]. Hominins, including those from *Homo naledi* (n=2) have modern humanlike proportions of the talar trochlea. Fossil hominins graphed here include: A.L. 288-1, StW 88, StW 363, TM 1517, U.W. 88-98 (MH2), Omo 323-76-898, OH 8, KNM-ER 813, KNM-ER 1464, KNM-ER 1476, and KNM-ER 5428. The box-and-whiskers plot shows the median (dark horizontal line), upper and lower quartiles (boxes), range (whiskers), and outliers (circles). Talar wedging ratio is the distal ML width of the trochlea divided by the proximal ML width of the trochlea multiplied by 100.

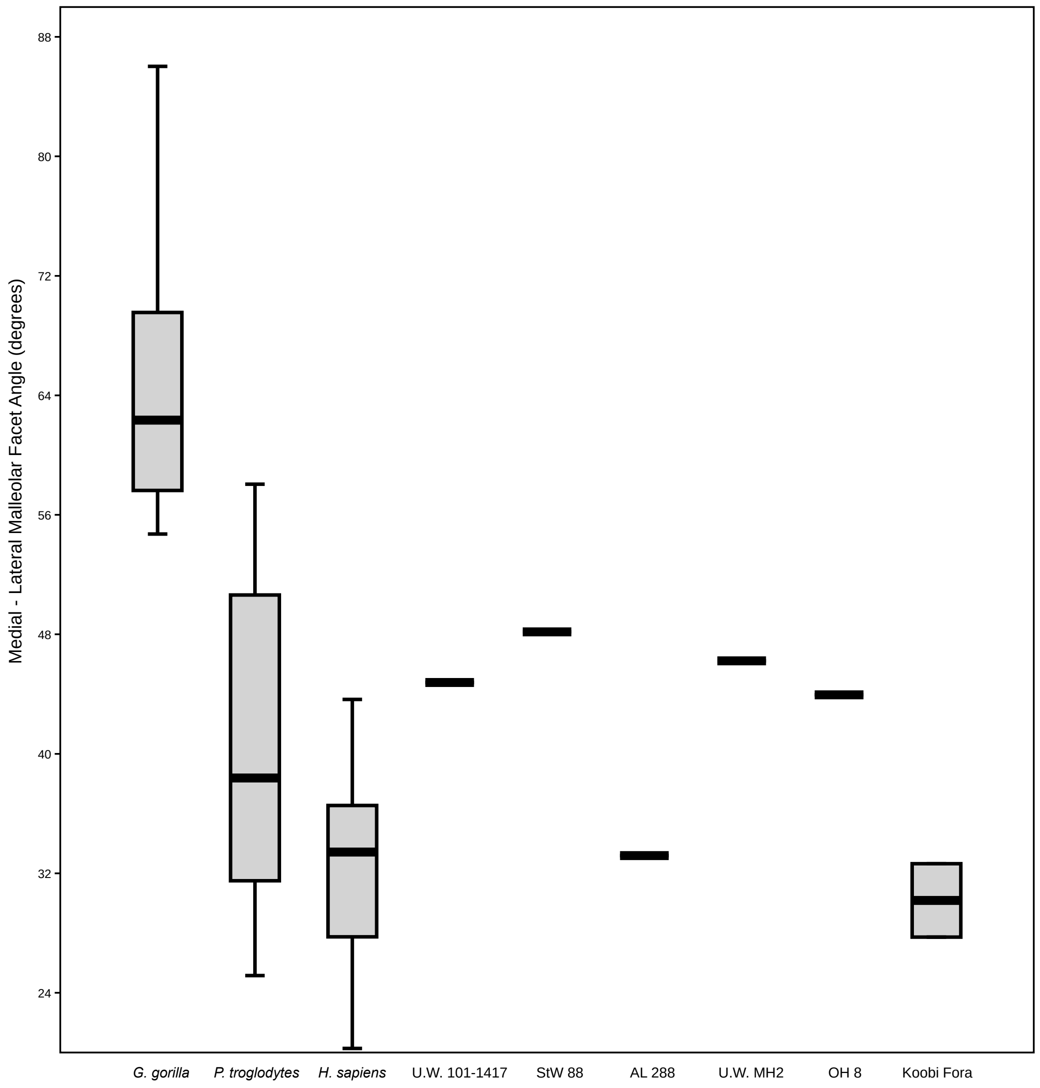

Supplementary Figure 4 | Angle between the medial and lateral malleoli of the talus. The angle is lowest in *H. sapiens*, and higher in the African Apes, especially in *Gorilla*. Given that the tali of *H. sapiens* are larger in size than those of *Pan*, this is unlikely to be related to size alone. Two tali from Koobi Fora (KNM-ER 1464 & 1476) and that of *Au. afarensis* (A.L. 288-1) fall well within the *H. sapiens* range of variation. OH 8, StW 88 and the tali of *Au. sediba* (U.W. 88-97; MH2) and *H. naledi* (U.W. 101-1417) fall just outside the human upper limit. The box-and-whiskers plot shows the median (dark horizontal line), upper and lower quartiles (boxes), range (whiskers), and outliers (circles).

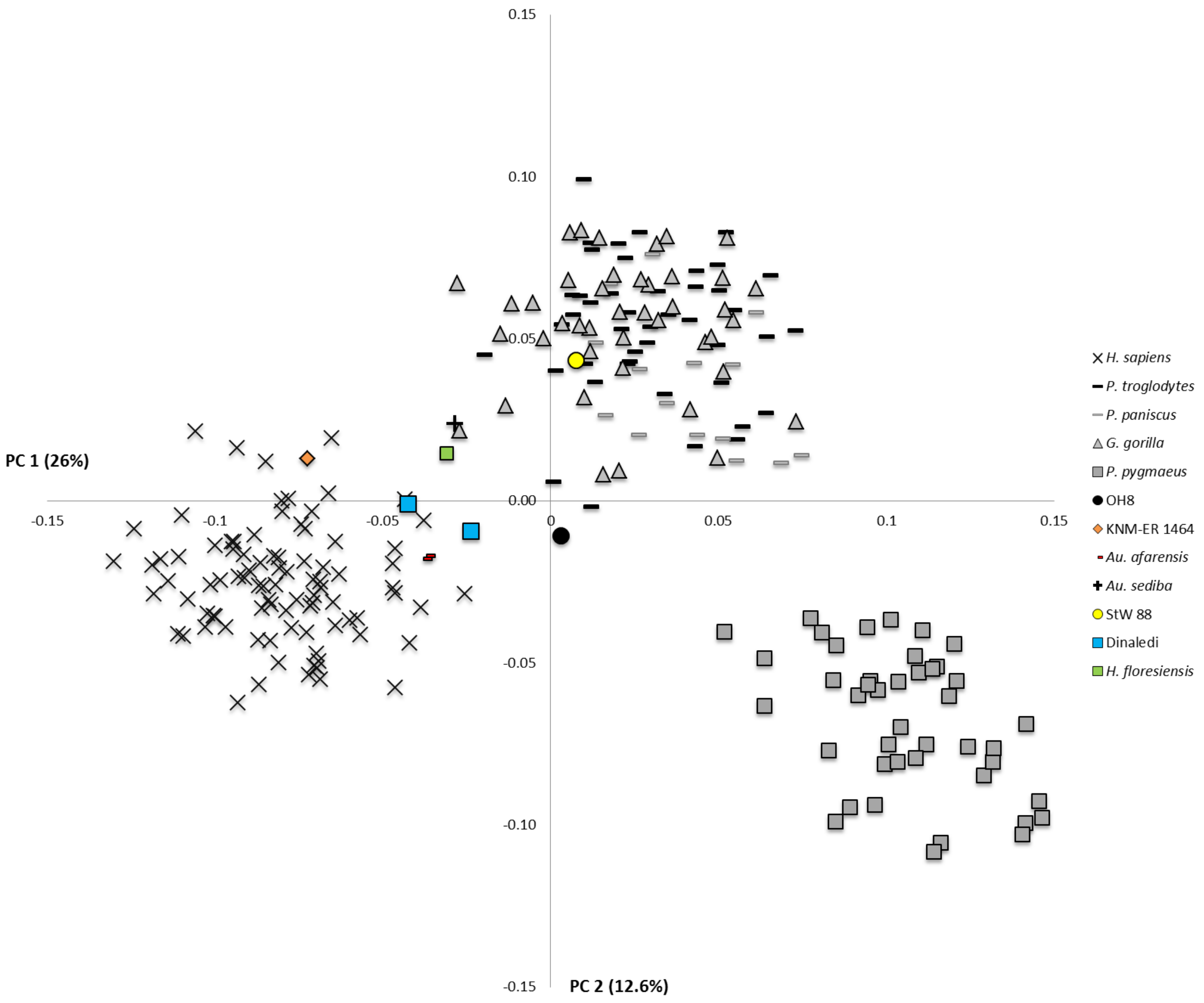

Supplementary Figure 5 | PCA of Generalized Procrustes analysis (GPA) aligned homologous 3D landmark coordinates for the talus. PC1 separates modern humans, *Au. afarensis* and KNM-ER 1464 from the extant great apes. StW 88 (*Au. africanus*) and MH2 (*Au. sediba*) fall within the great ape range (and outside that of the humans). OH 8 falls at the edge of the African ape range, but well outside that of humans. *H. floresiensis* is situated in the morphospace between the humans and the African great apes. The Dinaledi specimens (U.W. 101-148 & 1417) fall at the very edge of the human range of variation, with U.W. 101-1417 just outside it, and U.W. 101-148 just within it. On PC1 humans and the great apes separate due to the mediolaterly sloping, grooved trochlea of great apes, along with their more flared malleolar facets and more curved posterior calcaneal facet. Further anatomical details of this analysis (minus the Dinaledi specimens) can be found in Harcourt-Smith[2] and Jungers *et al.*[3].

.

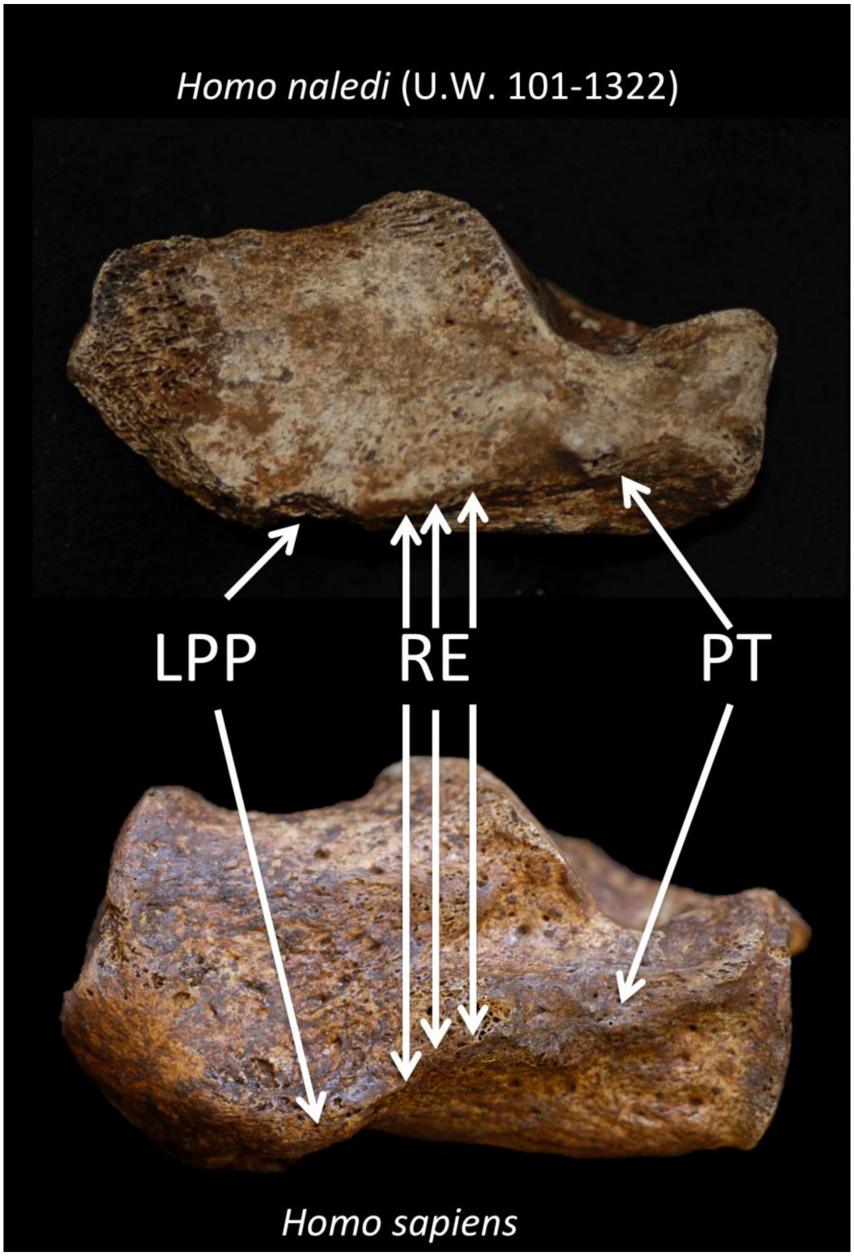

Supplementary Figure 6 | *H. naledi* calcaneus (U.W. 101-1322) compared to small-bodied modern *H. sapiens*. The two calcanei are shown in lateral view. Notice the similarly positioned and diminutive peroneal trochlea, plantarly deflecting retrotrochlear eminence, and plantarly positioned lateral plantar process on the two specimens.

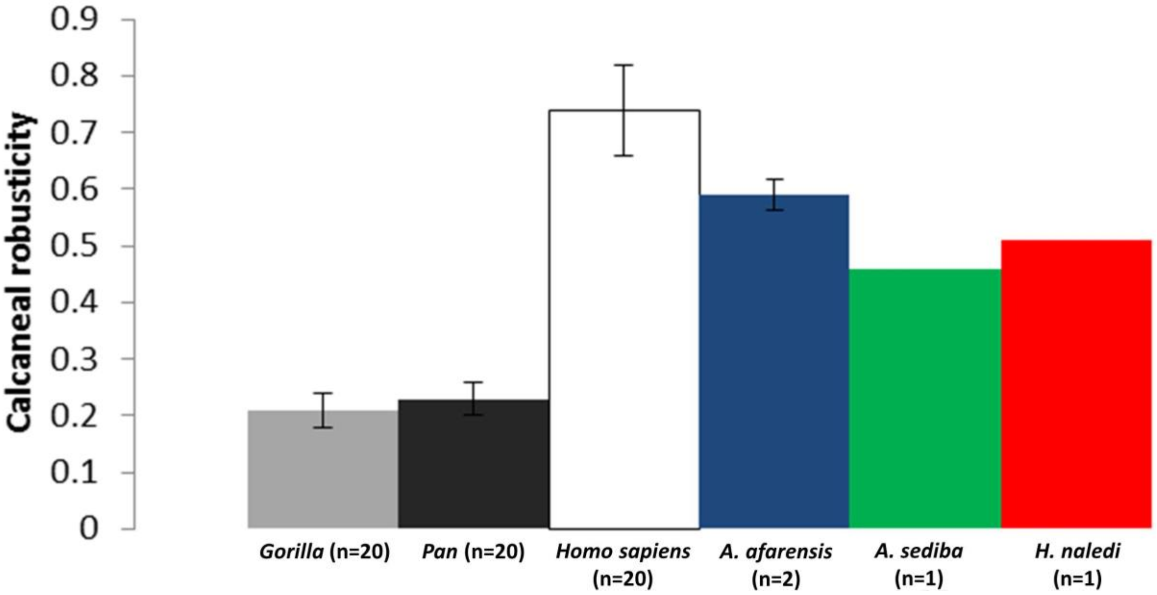

Supplementary Figure 7 | Calcaneal robusticity. Calcaneal robusticity was calculated as described in Latimer and Lovejoy[4] and Zipfel et al.[5] Relative to body mass, humans have a substantially more robust calcaneal tuber than that found in modern apes. The two calcanei from Hadar (body mass estimated from the A.L. 333-147 talus, which is from a similarly sized individual as A.L. 333-8 and A.L. 333-55) are quite robust as well, within two standard deviations of the modern human mean. The calcaneus from Malapa (U.W. 88-99), attributed to *Au. sediba*, is more gracile, 3.5 standard deviations away from the modern human mean, though substantially more robust than the calcaneus in modern apes. *Homo naledi* also has a gracile calcaneus, barely within three standard deviations of the modern human mean. However, unlike the calcaneus of *Au. sediba*, *Homo naledi* possesses the modern humanlike positioning of the plantar tubercles. Plotted are mean and standard deviation.

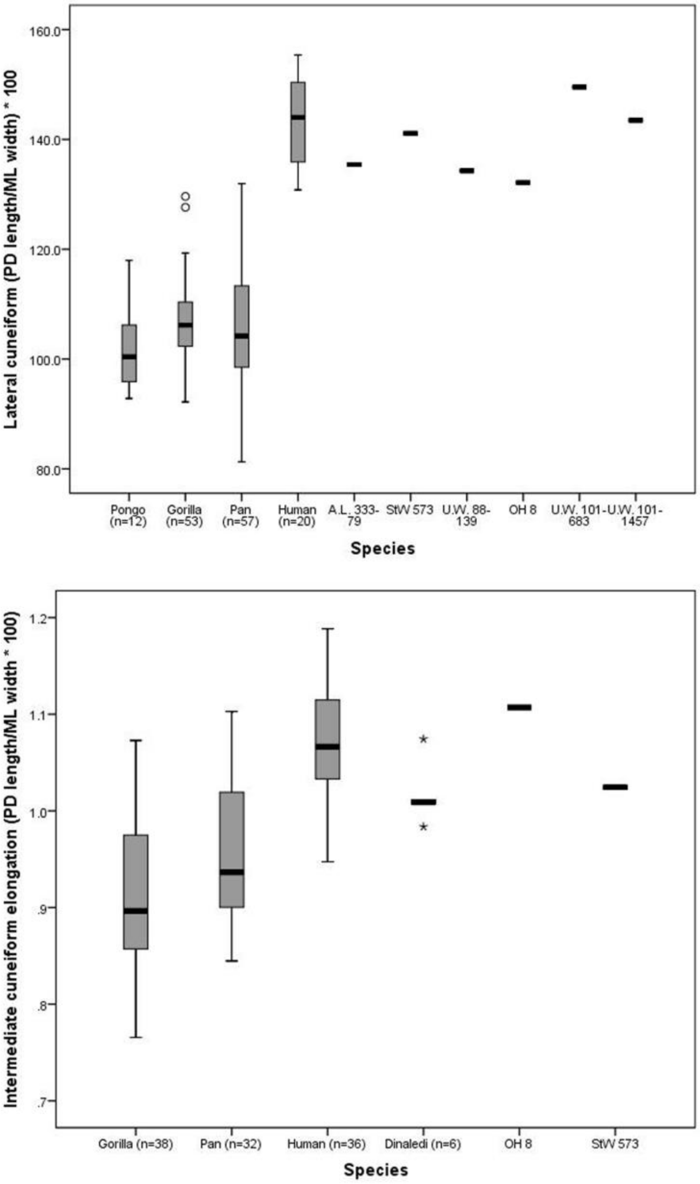

Supplementary Figure 8 | Relative intermediate and lateral cuneiform lengths.
Top. Lateral Cuneiform: bipedal hominins have an elongated tarsal region, quantified here using the dimensions of the lateral cuneiform. While extant great apes have squat lateral cuneiforms with roughly equal proximodistal and mediolateral dimensions, humans have a proximodistally elongated lateral cuneiform. All known fossil hominin lateral cuneiforms fit the human pattern and the Dinaledi hominins (n=2) are the most extreme in tarsal elongation. **Bottom.** Intermediate Cuneiform: compared to African apes, humans have proximodistally more elongated intermediate cuneiforms ($p<0.001$). This measurement is taken as a ratio of proximodistal length and mediolateral width. There are six intermediate cuneiforms from *Homo naledi.* They tend to be more elongate than those of chimpanzees, but not quite as long as those in most modern humans. However, there is one specimen—U. W. 101-1695—with proportions such as those found in the average human today. Box-and-whiskers plot shows the median (dark horizontal line), upper and lower quartiles (boxes), range (whiskers), and outliers (circles).

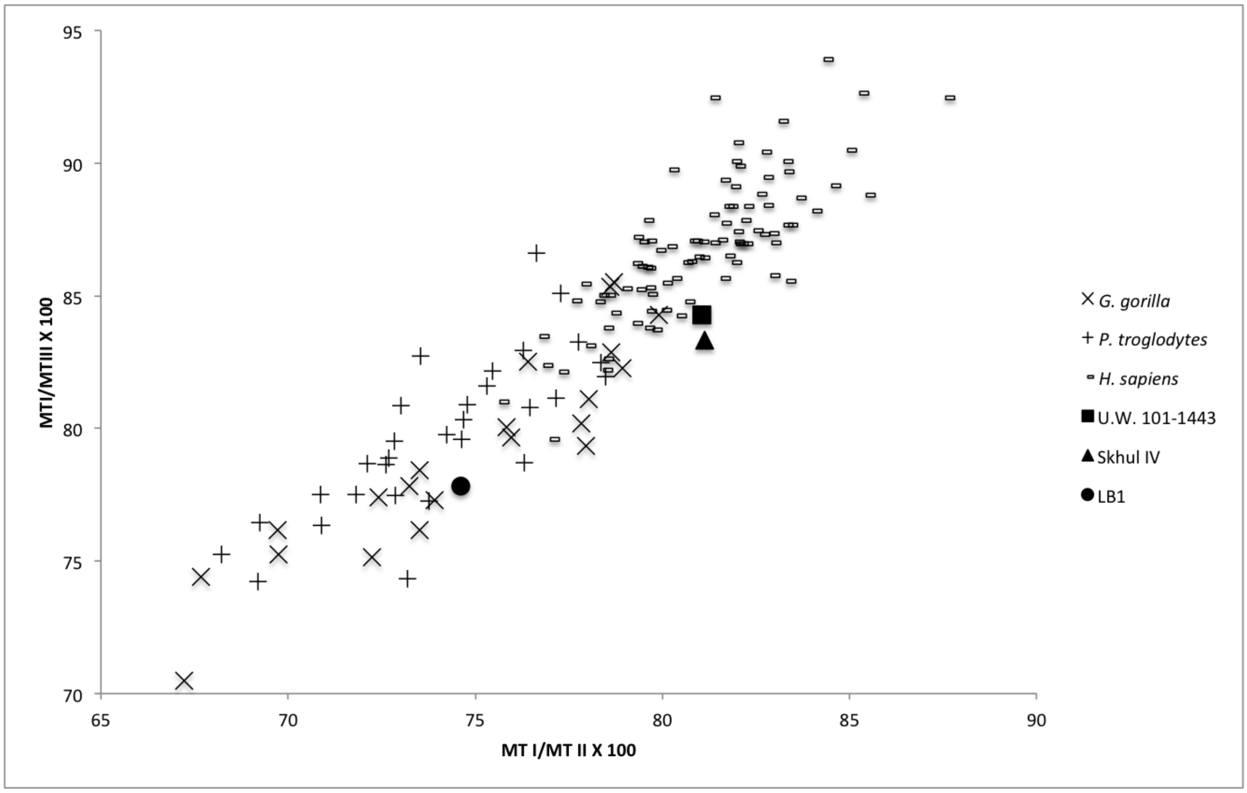

Supplementary Figure 9 | Length of the 1st metatarsal relative to the 2nd metatarsal. The hallux of *Pan* and *Gorilla* is relative short compared to that of *H. sapiens* (when compared to the 2nd and 3rd metatarsals). The hallux of *H. floresiensis* (LB1) is also short, and outside the human range[3], whilst those of *H. sapiens* (Skhul IV) and *H. naledi* (U.W.101-1443) are well within the human range and just outside that of the apes. The value for LB1 is taken from Jungers et al.[3].

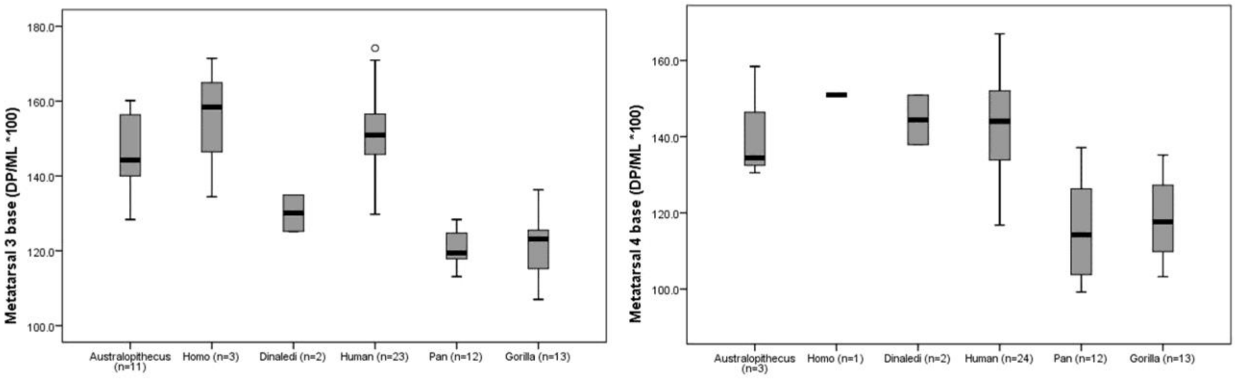

Supplementary Figure 10| 3rd and 4th metatarsal base height. Humans tend to have dorsoplantarly tall bases of their metatarsals. Compared to African apes, the human third and fourth metatarsals are dorsoplantarly tall, relative to their mediolateral width. The Dinaledi third metatarsals (U.W. 101-1035 and U.W. 101-1457) are intermediate between the values found in most modern humans and most African apes. Though there is some erosion plantarly and these values should be considered minimums, comparisons with complete human 3rd metatarsal indicate that the plantar base would not have gotten much deeper than what is preserved, if at all. The Dinaledi fourth metatarsals (U.W. 101-269 and U.W. 101-1456) have considerably taller bases, within the range of modern humans. *Australopithecus* third metatarsals include: A.L. 133-157, StW 238, StW 387, StW 388, StW 435, StW 477, StW 496, SKX 247, Omo F.511-16, KNM-ER 1500, and KNM-ER 1823. *Homo* third metatarsals include: OH 8, KNM-ER 803, and KNM-ER 997. *Australopithecus* fourth metatarsals are: A.L. 333-160, StW 485, and U.W. 88-22. The *Homo* fourth metatarsal is OH 8. Box-and-whiskers plot shows the median (dark horizontal line), upper and lower quartiles (boxes), range (whiskers), and outliers (circles).

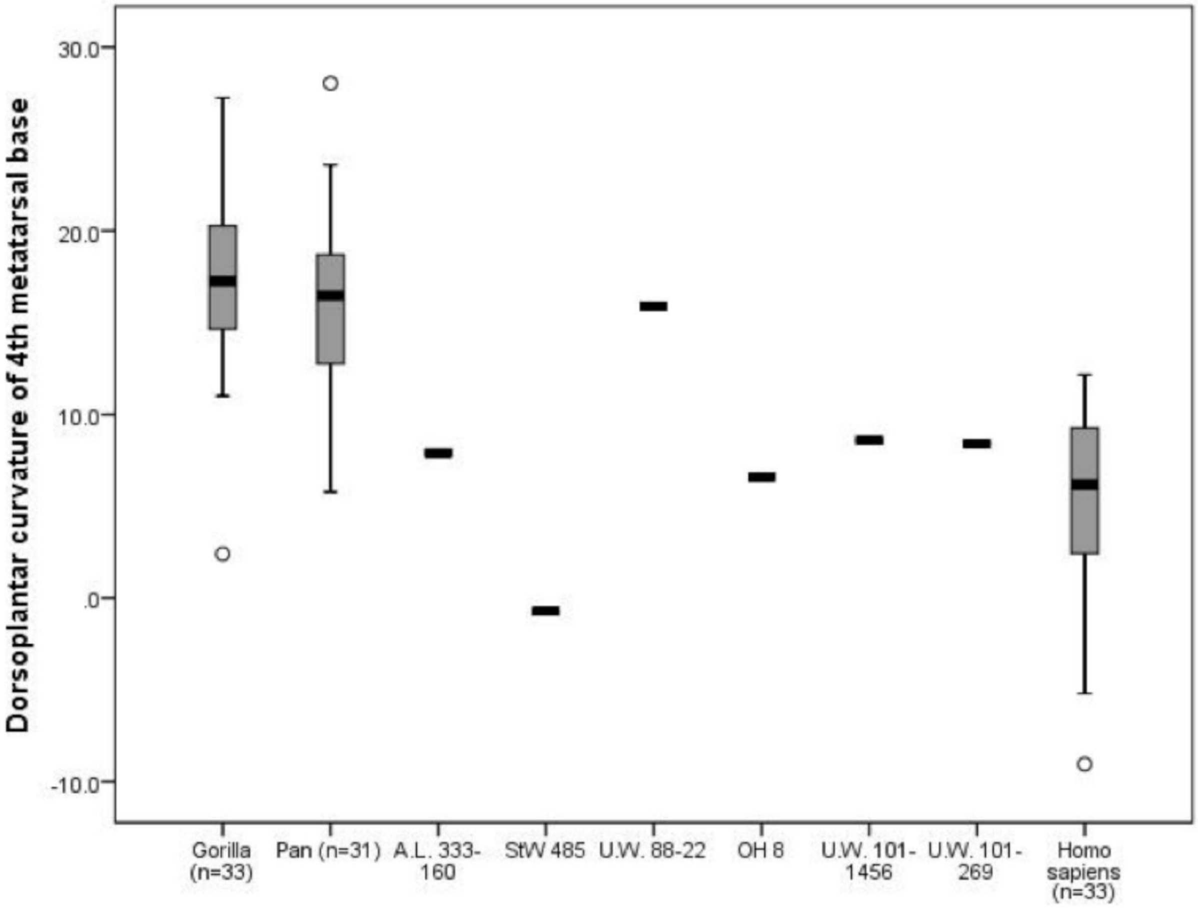

Supplementary Figure 11 | Dorsoplantar curvature of the 4th metatarsal base. 4th MT: African apes and other non-human primates have a flexible midfoot and regularly produce a "midtarsal break". A functional correlate of the midtarsal break is the dorsoplantar curvature of the base of the fourth metatarsal (measured as in DeSilva[6]). Humans tend to have a more rigid lateral midfoot and have a flatter base of the fourth metatarsal. All hominins, except for *Au. sediba*[7], fall within the range of modern humans, including the two complete fourth metatarsals from *Homo naledi.* The box-and-whiskers plot shows the median (dark horizontal line), upper and lower quartiles (boxes), range (whiskers), and outliers (circles).

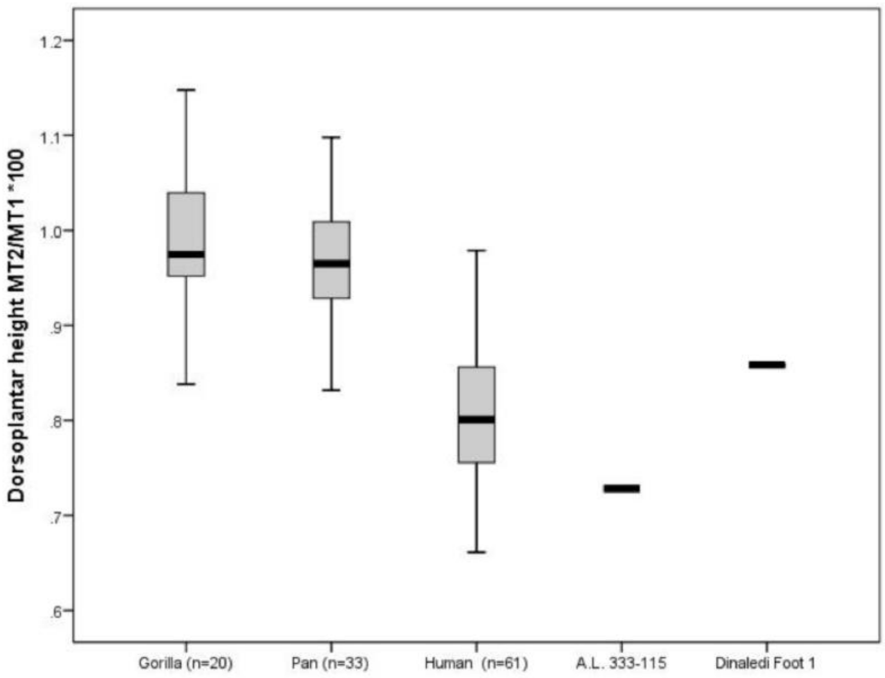

Supplementary Figure 12 | Relative 1st Metatarsal head dorsoplantar height. Relative to the dorsoplantar height of the second metatarsal, the first metatarsal head is larger in humans than in the African apes. This ratio may reflect the importance of the hallux in propulsion. Dinaledi Foot 1 has a relatively smaller hallucial head than most humans, though the range of modern human variation encompasses both *Au. afarensis* (A.L. 333-115) and *Homo naledi*. The box-and-whiskers plot shows the median (dark horizontal line), upper and lower quartiles (boxes), range (whiskers), and outliers (circles).

Supplementary Tables

There are 21 supplementary tables. SI Table 1 summarizes the entire Dinaledi pedal fossil sample to date and Supplementary Table 2 summarizes the main associated foot assemblages in the sample. Supplementary Tables 3-21 provide linear measurements, angles, indices and polynomial curve data for relevant specimens. In the tables that follow, PD=proximodistal; DP=dorsoplantar; ML=mediolateral; MT=metatarsal.

Supplementary Table 1. Pedal fossils of *Homo naledi.* from Dinaledi chamber, South Africa. *=immature

TARSALS		METATARSALS		PHALANGES	
Catalogue #	Element	Catalogue #	Element	Catalogue #	Element
**Talus**		**Metatarsal 1**		**Proximal phalanges**	
U.W. 101-080	Left talus*	U.W. 101-244	Left MT1*	U.W. 101-082	Left hallucial
U.W. 101-148/149	Left talus	U.W. 101-496	Left MT1	U.W. 101-504	Left
U.W. 101-520	Left talus	U.W. 101-1019	Left MT1	U.W. 101-725	Unsided head and shaft
U.W. 101-910	Left talus*	U.W. 101-1443	Right MT1	U.W. 101-976	Unsided
U.W. 101-1031	Left talus	U.W. 101-1530	Right MT1	U.W. 101-1013	Left
U.W. 101-1215	Left talus fragment	U.W. 101-1499	Right MT1 epiphysis*	U.W. 101-1024	Left hallucial
U.W. 101-1417	Right talus			U.W. 101-1034	Left
U.W. 101-1623	Right talus*	**Metatarsal 2**		U.W. 101-1148	Unsided
**Calcaneus**		U.W. 101-459/461	Right MT2	U.W. 101-1395	Unsided
U.W. 101-724	Left calcaneus	U.W. 101-1022	Left MT2	U.W. 101-1419	Right hallucial
U.W. 101-907	Left calcaneua*	U.W. 101-1458	Right MT2	U.W. 101-1441	Unsided
U.W. 101-1322	Right calcaneus	U.W. 101-1499	Right MT2*	U.W. 101-1442	Hallucial
U.W. 101-1662	Right calcaneal frag.*	**Metatarsal 3**		U.W. 101-1452	Hallucial
**Navicular**		U.W. 101-552	Left MT3	U.W. 101-1557	Unsided
U.W. 101-623	Right nav. fragment	U.W. 101-1035	Left MT3	U.W. 101-1657	Unsided*
U.W. 101-811	Left navicular	U.W. 101-1457	Right MT3		
U.W. 101-910	Left navicular*	U.W. 101-1500	Right MT3*		
U.W. 101-997	Right navicular*	**Metatarsal 4**		**Intermediate phalanges**	**Distal phalanges**
U.W. 101-1030	Left navicular	U.W. 101-248	Left MT4*	U.W. 101-550	U.W. 101-988
U.W. 101-1562	Right navicular	U.W. 101-269	Right MT4	U.W. 101-661	U.W. 101-1010
		U.W. 101-1368	Right MT4*	U.W. 101-988	U.W. 101-1526
**Medial cuneiform**		U.W. 101-1456	Right MT4	U.W. 101-1042	U.W. 101-1550
				U.W. 101-1399	U.W. 101-1551
U.W. 101-1039	Left med. cun.	**Metatarsal 5**		U.W. 101-1438	U.W. 101-1576
U.W. 101-1062	Left med. cun.	U.W. 101-518	Right MT5	U.W. 101-1484	
U.W. 101-1535	Left med. cun.	U.W. 101-1412	Right MT5	U.W. 101-1549	
**Intermediate cuneiform**		U.W. 101-1439	Right MT5	U.W. 101-1575	
U.W. 101-1242	Right juv. int. cun.*	**Metatarsal fragments**		U.W. 101-1587	**Phalangeal fragments**
U.W. 101-1457	Right int. cun.	U.W. 101-497	Metatarsal shaft	U.W. 101-1591	
U.W. 101-1534	Left int. cun.	U.W. 101-750	Metatarsal shaft	U.W. 101-1594	U.W. 101-884
U.W. 101-1618	Left int. cun	U.W. 101-801	Metatarsal head fragment	U.W. 101-1625	U.W. 101-1118
U.W. 101-1682	Left int. cun.*.				U.W. 101-1589
U.W. 101-1695	Right int. cun.	U.W. 101-869	Metatarsal head fragment		U.W. 101-1592
					U.W. 101-1595
		U.W. 101-1437	2 metatarsal shaft fragments		AU.W. 101-1598
**Lateral cuneiform**		U.W. 101-1444	Metatarsal shaft	**Sesamoid**	
U.W. 101-683	Left adult lat. cun.	U.W. 101-1513	Metatarsal shaft	U.W. 101-1553	
U.W. 101-1698	Right adult lat. cun.	U.W. 101-1559	Metatarsal shaft		
U.W. 101-1734	Left lat. cun.	U.W. 101-1585	Metatarsal shaft		
**Cuboid**					
U.W. 101-487	Right cuboid*				
U.W. 101-1023	Left cuboid				
U.W. 101-1418	Right cuboid				

Supplementary Table 2. Associated pedal elements of *Homo naledi* from Dinaledi chamber, South Africa.

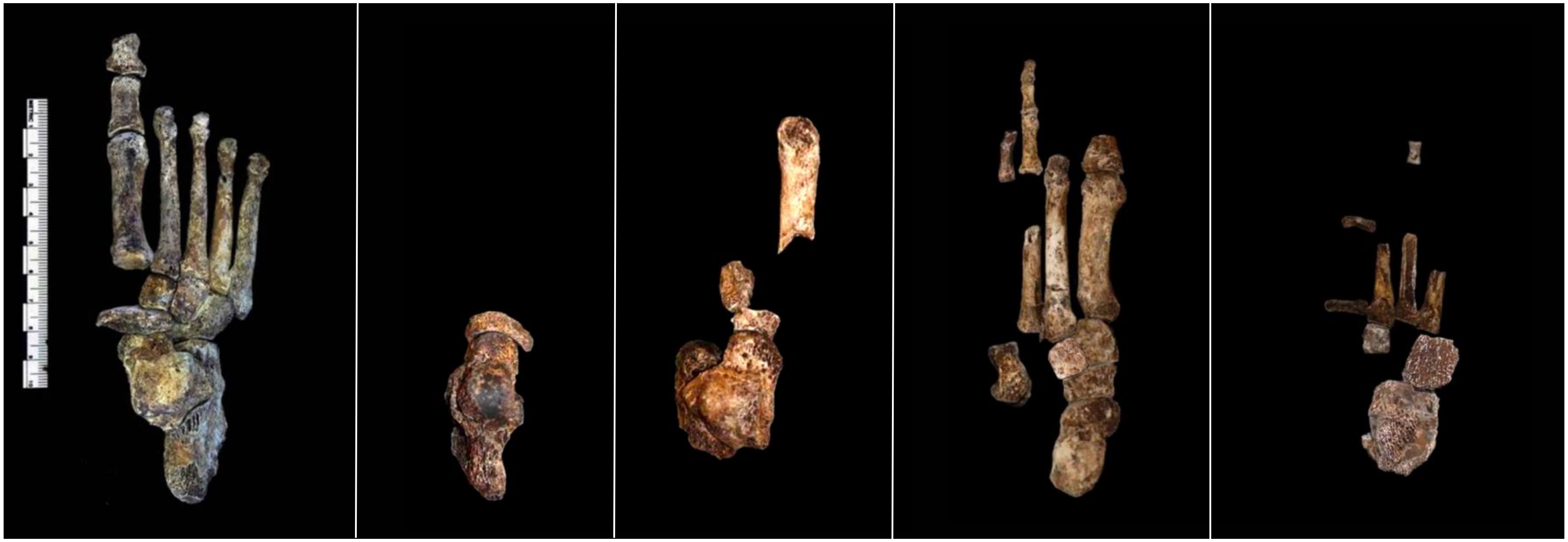

Foot 1. Adult right		Foot 2. Immature left		+Foot 3. Adult left		Foot 4. Adult left		*Foot 5. Immature right	
Catalogue #	Element	Catalogue #	Element	Catalogue #	Element	Catalogue #	Element	Catalogue #	Element
U.W. 101-1322	Calcaneus	U.W. 101-907	Calcaneus	U.W. 101-496	Metatarsal 1	U.W. 101-1019	Metatarsal 1	U.W. 101-1368	Metatarsal 4
U.W. 101-1417	Talus	U.W. 101-910	Talus; Navicular	U.W. 101-520	Talus	U.W. 101-1022	Metatarsal 2	U.W. 101-1483 and U.W. 101-1484	Middle phalanx; Proximal hallucial phalanx
U.W. 101-1418	Cuboid			U.W. 101-683	Lateral cuneiform	U.W. 101-1023	Cuboid	U.W. 101-1499	Metatarsal 1 base; Metatarsal 2
U.W. 101-1419	Proximal hallucial phalanx			U.W. 101-724	Calcaneus	U.W. 101-1024	Proximal hallucial phalanx	U.W. 101-1500	Metatarsal 3
U.W. 101-1439	Metatarsal 5			U.W. 101-811	Navicular	U.W. 101-1030	Navicular	U.W. 101-487	Cuboid
U.W. 101-1443	Metatarsal 1					U.W. 101-1031	Talus	U.W. 101-1242	Intermediate cuneiform
U.W. 101-1456	Metatarsal 4					U.W. 101-1034	Proximal phalanx	U.W. 101-1623	Talus
U.W. 101-1457	Metatarsal 3; Int. cuneiform					U.W. 101-1035	Metatarsal 3	U.W. 101-1662	Calcaneus
U.W. 101-1458	Metatarsal 2					U.W. 101-1039	Medial cuneiform		
U.W. 101-1551	Distal hallucial phalanx					U.W. 101-1010	Distal phalanx		
U.W. 101-1553	FHB sesamoid					U.W. 101-1013	Proximal phalanx		
U.W. 101-1562	Navicular					U.W. 101-1042	Middle phalanx		
U.W. 101-1698	Lateral cuneiform					U.W. 101-1618	Intermediate cuneiform		

Note: We define a "foot" by the presence of at least three associated elements. Additional specimens are associated (i.e. U.W. 101-244 [MT 1] & U.W. 101-248 [MT 4], and U.W. 101-1534 [intermediate cuneiform] & U.W. 101-1535 [medial cuneiform]) but are not listed here as "feet". Feet 1, 2, and 4 are supported by both anatomical congruence between individual elements and by taphonomic association. Specimens shaded are likely, but not definitively, associated elements. Precise digit assignment of the lateral phalanges in the foot photographs above is tentative.

+Adult foot 3 is based on morphological and size congruity between the individual elements. However, they were collected in a widely dispersed region and therefore this foot is not as certain on taphonomic grounds.

*Immature foot 5 is based on morphological and size congruity between the elements. Specimens U.W. 101-467, -1623, and -1662 were collected together and are almost certainly associated. The association of these tarsals with the metatarsals and phalanges is questionable.

Supplementary Table 3. Talus: linear measurements (in mm)

Specimen	Total size of bone			Trochlea dimensions				Neck length	Head		Fibular facet flaring
	PD	ML	DP	PD	Distal ML	Mid ML	Proximal ML	PD	DP	ML	ML
U.W. 101-80	34.5 (min)	23.2 (min)	19.2	17.1	NA	14.2	NA	7.4*	10.1 (min)	15.7*	NA
U.W. 101-148/149	43.4	39.0	25.7	26.4	22.4	20.6	19.5	8.9	18.4	23.9*	8.6
U.W. 101-520	40.8	35.6	22.0*	26.2	19.7	18.3*	NA	8.1	13.4 (min)	21.3*	7.8
U.W. 101-910	34.9	NA	19.4	19.0	NA	15.4 (min)	NA	10.7	14.0	19.7	NA
U.W. 101-1031	NA	NA	NA	NA	NA	18.5	NA	8.5	NA	20.9 (min)	5.5 (min)
U.W. 101-1417	38.4	34.5	19.0	21.5	19.6	18.4	17.1	9.9	14.3	20.7*	6.6 (min)
U.W. 101-1623	NA	NA	NA	NA	13.8	NA	NA	8.5	11.5*	14.8*	NA

*=approximate; (min)= minimum; NA= unmeasurable

Supplementary Table 4. Talus: angular measurements (°). After Day and Wood[8]

Specimen	Horizontal angle of the head/neck	Angle of torsion of the head/neck	Angle of inclination (declination) of the head/neck
U.W. 101-80	NA	21	15
U.W. 101-148/149	25	45	14
U.W. 101-520	20*	35*	18*
U.W. 101-910	NA	42*	NA
U.W. 101-1031	NA	34*	NA
U.W. 101-1417	26	37	10

*=approximate; NA= unmeasurable

Supplementary Table 5. Calcaneus: linear measurements (in mm)

Specimen	Total length	Minimum tuber dimensions		Proximal talar facet		Distal talar facet		Sustentaculum thickness	FHL groove	Cuboid facet	
	PD	DP	ML	PD	ML	PD	ML	DP	ML	DP	ML
U.W. 101-724	NA	NA	NA	17.6 (min)	15.4*	NA	NA	9.8	3.9	NA	18.9
U.W. 101-907	47.7*	22.0*	NA	20.2	NA	18.9 (min)	9.5	7.0*	4.0*	15.4	16.7
U.W. 101-1322	57.0	25.0*	19.0*	20.8	14.4	19.6	9.5	8.8	4.2	14.8	19.5
U.W. 101-1662	NA	NA	NA	16.6	11.6	NA	NA	NA	NA	NA	NA

*=approximate; (min)= minimum; NA= unmeasurable

Supplementary Table 6. Navicular: linear measurements (in mm).

Specimen	Total		PD thickness		Talar facet		Lat. cun. facet		Int. cun. facet		Med. cun. facet	
	ML	DP	Medial	Lateral	ML	DP	ML	DP	ML	DP	ML	DP
U.W. 101-623	NA	NA	8.7*	8.5*	NA	NA	NA	NA	NA	NA	NA	NA
U.W. 101-811	NA	NA	8.4	7.9	NA	NA	6.5	6.6 (min)	6.5 (min)	7.3*	NA	NA
U.W. 101-910	23.5	14.2	8.3	6.8	20.4	12.8	NA	NA	NA	NA	NA	NA
U.W. 101-997	NA	NA	NA	5.7*	15.3*	11.4	6.4*	4.2*	5.6*	8.3*	NA	NA
U.W. 101-1030	NA	NA	11.9	8.9	NA	13.5	NA	NA	9.7	9.5	7.9 (min)	7.9
U.W. 101-1562	27.9*	14.7*	9.4	6.5	18.9	12.3	8.2	7.0	6.4	8.3	NA	9.1

*=approximate; (min)= minimum; NA= unmeasurable

Supplementary Table 7. Medial cuneiform: linear measurements (in mm).

Specimen	Total dimensions			Int. cun. facet		Navicular facet		MT2 facet		MT1 facet	
	DP	PD	ML	DP	PD	DP	ML	DP	PD	DP	ML
U.W. 101-1039	22.7*	NA	11.9*	NA	9.1*	NA	NA	NA	NA	NA	NA
U.W. 101-1062	24.5	17.3*	11.4 (min)	13.0*	9.8	14.1*	9.2*	NA	NA	NA	NA
U.W. 101-1535	24.2	20.2	14.2	NA	NA	NA	NA	6.2	5.4	19.3	12.6

*=approximate; (min)= minimum; NA= unmeasurable

Supplementary Table 8. Intermediate cuneiform: linear measurements (in mm).

Specimen	Total dimensions			Med. cun. facet		Lat. cun. facet		Navicular facet		MT2 facet	
	DP	PD	ML	DP	PD	DP	PD	DP	ML	DP	ML
U.W. 101-1242	13.8	10.8	10.7	NA	NA	NA	NA	12.0*	9.3	12.5 (min)	8.9
U.W. 101-1457	14.0	12.1	12.0	NA	NA	6.6	7.6	13.0*	11.3	13.4	9.6
U.W. 101-1534	18.3	12.4	12.3	9.5*	11.8	7.9	8.3	15.2	12.6	14.0*	9.6
U.W. 101-1618	15.0	12.0	12.2	5.9	9.0	6.5	10.3	12.7	10.1	NA	NA
U.W. 101-1682	10.2*	8.8	8.7	5.5*	7.9	NA	8.2*	10.0*	6.6	NA	NA
U.W. 101-1695	13.3 (min)	13.0	12.1	NA	NA	6.7 (min)	8.5	12.8 (min)	11.0	NA	11.5

*=approximate; (min)= minimum; NA= unmeasurable

Supplementary Table 9. Lateral cuneiform: linear measurements (in mm).

Specimen	Total dimensions			Cuboid facet		MT 4 facet	Int. cun. facet		Navicular facet		MT3 facet	
	DP	PD	ML	DP	PD	PD	DP	PD	DP	ML	DP	ML
U.W. 101-683	15.7	16.0	10.7	7.1	10.5	2.7	6.2	6.0*	7.4*	7.2*	14.1	9.5
U.W. 101-1698	14.6	16.5	11.5	7.3	10.9*	3.8	8.4	7.7	8.8*	7.8*	14.3*	10.4
U.W. 101-1734	NA	13.9*	9.5*	NA	NA	NA	NA	NA	9.3*	6.7*	NA	NA

*=approximate; (min)= minimum; NA= unmeasurable

Supplementary Table 10. Cuboid: linear measurements (in mm).

Specimen	Total dimensions			Proximal projection of beak	Lat. cun. facet		Calcaneal facet		MT4 facet		MT5 facet	
	DP	PD	ML	PD	DP	PD	DP	ML	DP	ML	DP	ML
U.W. 101-467	NA	19.8 (min)	15.3 (min)	NA	NA	NA	NA	NA	NA	NA	NA	NA
U.W. 101-1023	17.5	23.1	NA	3.3	NA	NA	NA	NA	NA	NA	NA	NA
U.W. 101-1418	18.1	24.4	NA	5.8	7.0*	10.5*	17.1	14.9	12.5	7.9	7.8	9.0

*=approximate; (min)= minimum; NA= unmeasurable

Supplementary Table 11. Metatarsal 1: linear measurements (in mm).

Specimen	Total length	Base dimensions		+Head dimensions		Midshaft dimensions		Med. cun. facet		MT2 facet	
	PD	DP	ML	DP	ML	DP	ML	DP	ML	DP	PD
U.W. 101-244	38.9	14.0	9.0*	13.5	9.5	8.9	8.3	NA	NA	NA	NA
U.W. 101-496	NA	NA	NA	15.1 (min)	14.2	10.6	11.9	NA	NA	NA	NA
U.W. 101-1019	55.1*	18.2 (min)	12.7 (min)	15.6*	13.8*	10.3	10.9	NA	NA	6.1	7.0
U.W. 101-1443	50.0	21.1	13.5	13.0	13.4	10.0	10.5	19.8	12.6	6.6*	3.0*
U.W. 101-1530	49.0 (min)	22.2	13.3	NA	NA	10.3*	11.1*	18.2	11.0	NA	NA

+= measurement does not include plantar cornua (though difficult to discern on immature specimen 101-244); *=approximate; (min)= minimum; NA= unmeasurable

Supplementary Table 12. Metatarsal 2: linear dimensions (in mm).

Specimen	Total length	Base dimensions		Head dimensions		Midshaft dimensions		Int. cun. facet		MT3 facet		Lat. cun. facet		Med. cun. facet	
	PD	DP	ML	DP	ML	DP	ML	DP	ML	DP	PD	DP	PD	DP	PD
U.W. 101-459/461	58.0*	12.6 (min)	10.1	NA	NA	6.9	6.6	NA	NA	NA	NA	NA	NA	NA	NA
U.W. 101-1022	67.5	13.6 (min)	13.2 (min)	NA	NA	7.2	7.6	NA	11.8	4.5	6.9	6.3	3.2	NA	NA
U.W. 101-1458	63.4	13.6 (min)	11.5	11.5	NA	6.8	6.3	11.2*	10.5	6.1	6.7	6.0	4.0	6.3*	5.1*
U.W. 101-1499	NA	11.3	9.7	NA	NA	NA	NA	NA	8.0*	4.1	6.3	3.4*	3.4*	NA	NA

*=approximate; (min)= minimum; NA= unmeasurable

Supplementary Table 13. Metatarsal 3: linear dimensions (in mm).

Specimen	Total length	Base dimensions		Head dimensions		Midshaft dimensions		Lat. cun. facet		MT2 facet		MT4 facet	
	PD	DP	ML	DP	ML	DP	ML	DP	ML	DP	PD	DP	PD
U.W. 101-552	60.0*	11.2 (min)	11.9	NA	NA	6.8	7.0	NA	10.8 (min)	NA	NA	6.9*	9.4*
U.W. 101-1035	NA	14.3	10.6	NA	NA	6.9*	5.9*	NA	NA	4.9*	NA	NA	NA
U.W. 101-1457	60.4	13.9	11.1	10.2 (min)	NA	6.2	5.8	13.8	10.0*	5.7*	6.0*	6.5	8.9
U.W. 101-1500	NA	10.4 (min)	9.2	NA	NA	NA	NA	9.3 (min)	8.6	4.9	6.1	NA	NA

*=approximate; (min)= minimum; NA= unmeasurable

Supplementary Table 14. Metatarsal: 4 linear dimensions (in mm).

Specimen	Total length	Base dimensions		Head dimensions		Midshaft dimensions		Cuboid facet		MT3 facet		Lat. cun. facet	MT5 facet	
	PD	DP	ML	DP	ML	DP	ML	DP	ML	DP	PD	PD	DP	PD
U.W. 101-248	NA	9.2	7.2 (min)	NA	NA	NA	NA	NA	NA	NA	NA	NA	NA	NA
U.W. 101-269	59.0	13.1	9.5	8.8 (min)	7.1	7.5	6.5	12.1	8.9	6.6	4.5	4.9	6.2*	8.4*
U.W. 101-1368	NA	11.3*	7.6*	NA	NA	NA	NA	NA	NA	5.2	4.1 (min)	3.3	5.6	6.8
U.W. 101-1456	59.0	16.0	10.6	12.6*	7.6*	7.6	6.7	14.3	9.7	7.3	8.1	2.9	5.9*	8.9

*=approximate; (min)= minimum; NA= unmeasurable

Supplementary Table 15. Metatarsal 5: linear dimensions (in mm).

Specimen	Total length	Base dimensions		Head dimensions		Midshaft dimensions		Cuboid facet		MT4 facet	
	PD	DP	ML	DP	ML	DP	ML	DP	ML	DP	PD
U.W. 101-518	NA	10.0	15.0	NA	NA	5.3*	8.4*	9.7	10.6	10.0	8.9
U.W. 101-1412	NA	NA	NA	NA	NA	6.1*	8.0*	NA	NA	9.7	7.2
U.W. 101-1439	60.2	8.3	13.1	NA	NA	5.4	8.1	8.1	9.6	8.0*	8.5

*=approximate; (min)= minimum; NA= unmeasurable

Supplementary Table 16. Proximal phalanx: linear dimensions (in mm).

Specimen	Length	DP base	ML base	DP head	ML head	DP shaft	ML shaft
U.W. 101-082	28.6	9.4 (min)	11.8 (min)	6.6	11.1 (min)	7.7	9.8
U.W. 101-504	25.5	9.7	8.6 (min)	4.9*	8.1	6.3	6.5
U.W. 101-725	22.8 (min)	8.3*	6.8*	4.3 (min)	7.4	5.1	5.8
U.W. 101-976	23.6*	9.9	8.3 (min)	4.2 (min)	6.6 (min)	5.3	5.9
U.W. 101-1013	22.4	8.4	7.1 (min)	4.9	6.4	5.1	5.5
U.W. 101-1024	NA	12.4 (min)	15.4	NA	NA	NA	NA
U.W. 101-1034	19.4	NA	NA	3.5	5.9	4.3	4.3
U.W. 101-1148	21.5	8.9	7.9	4.5	6.3	5.5	4.9
U.W. 101-1395	21.6	8.6	8.4	4.6	6.8	5.6	5.2
U.W. 101-1395+	NA	8.1*	NA	NA	NA	4.4*	4.9*
U.W. 101-1419	NA	10.2	12.5	NA	NA	5.2	8.8
U.W. 101-1441	23.3	9.5	8.5	4.7	7.2	5.9	5.4
U.W. 101-1442	NA	12.1	13.3	NA	NA	7.0	9.9
U.W. 101-1452	24.7	8.8	10.5	7.0	10.9	5.7	8.0
U.W. 101-1557	NA	NA	NA	3.3	5.1	NA	NA
U.W. 101-1657	NA	NA	NA	3.3	3.0	4.0	4.5

*=approximate; (min)= minimum; NA= unmeasurable; += two proximal phalanges were recovered together and are both catalogued as U.W. 101-1359.

Supplementary Table 17. Intermediate phalanx: linear dimensions (in mm).

Specimen	Length	DP base	ML base	DP head	ML head	DP shaft	ML shaft
U.W. 101-550	12.7	5.6	NA	4.2	7.1	3.7	5.5
U.W. 101-661	10.6	4.6	6.8	3.9	5.5	3.1	4.5
U.W. 101-988	11.5	6.1 (min)	6.8	3.5	6.6	3.5	5.1
U.W. 101-1042	11.9	5.9	6.6	3.8	6.1	3.6	4.4
U.W. 101-1399	16.7	6.9	9.3	4.3	7.1	4.4	6.0
U.W. 101-1438	10.8	6.1	7.5	4.1	6.8	3.6	6.0
U.W. 101-1484	8.6	4.7	5.9	3.5	5.8	3.7	4.7
U.W. 101-1549	8.3	5.9	6.8	3.8	6.0	3.0	5.0
U.W. 101-1575	NA	NA	NA	3.6	6.0	NA	NA
U.W. 101-1587	5.5	6.2	8.0	3.2	6.2	2.9	6.3
U.W. 101-1591	NA	6.1	7.0	NA	NA	NA	NA
U.W. 101-1594	6.3	3.1*	4.4*	2.1*	4.3*	2.5	3.8
U.W. 101-1625	NA	NA	NA	2.9	5.2	3.2	5.1

*=approximate; (min)= minimum; NA= unmeasurable

Supplementary Table 18. Distal phalanx: linear measurements (in mm).

Specimen	Length	DP base	ML base	DP head	ML head	DP shaft	ML shaft
U.W. 101-988	8.2	4.4	5.7	2.6	4.4	3.2	4.0
U.W. 101-1010	8.6	5.0	6.5	2.8	4.6	3.2	4.1
U.W. 101-1526	7.0	5.2	5.7	2.3	4.1	3.0	3.5
U.W. 101-1550	7.9	4.7	6.8	2.4	4.1	3.6	3.5
U.W. 101-1551	15.5	5.7 (min)	14.9 (min)	5.3	10.3 (min)	6.5	9.5
U.W. 101-1576	4.9	5.8	6.5	3.5	5.9	4.3	6.1

Supplementary Table 19. Dorsal canting angle of Dinaledi proximal pedal phalanges

**Proximal**	**Dorsal canting angle (°)**
U.W. 101-082	107.4
U.W. 101-504	113.2
U.W. 101-1013	107.0
U.W. 101-1395	115.3
U.W. 101-1441	113.4
U.W. 101-1452	97.5

Supplementary Table 20. Proximal pedal phalanges used in the phalangeal curvature analyses

Proximal	1st polynomial coefficient
U.W. 101-1034	.09516
U.W. 101-1557	.08633
U.W. 101-1589	.04221
U.W. 101-1598	-.03054
U.W. 101-1657	.03730
U.W. 101-504	.07595
U.W. 101-725	.07827
U.W. 101-976	.02883
U.W. 101-1148	.02540
U.W. 101-1395	.06094
U.W. 101-1441	.08885
**Sample Mean**	**0.0535180**

The 1st polynomial coefficient (A) from the polynomial function (y=Ax2+BX+C) for each Dinaledi specimen. The first coefficient (A) expresses the nature and degree of the longitudinal curvature whereas the second (B) and third (C) reflect aspects of the orientation of that curve with respect to the rest of the element (i.e. element rotation, element position in 2-dimensional space). Only the first coefficient (A) was used in the analysis of pedal phalangeal curvature.

Supplementary Table 21. Average Power arm/Load arm ratio in *Homo naledi* (Foot 1), humans and apes. After Schultz[9].

	Specimens	(Power arm/ Load arm)*100
Homo naledi	**1**	**40.8**[a]
Homo sapiens	25	39.4
Gorilla	20	46.1
Pan	20	28.2
Pongo	9	19.6
Symphalanges	5	22.7
Hylobates	18	18.2

[a]Power arm = 40mm; Load arm = 98mm

The ratio between the power arm of the foot (the distance between the centre of the trochlea of the talus to the calcaneal tuberosity) and the load arm (the distance between the centre of the talar trochlea to the head of the third metatarsal) of Foot 1 falls within the range of modern humans.

Supplementary Note 1

Anatomical descriptions of the pedal elements of *H. naledi*.

Below are descriptions of the pedal remains from *Homo naledi*. The descriptions are arranged from the rearfoot (talus and calcaneus), through the midfoot (cuneiforms, navicular, cuboid), to the forefoot (metatarsals and phalanges). The descriptions are primarily qualitative; most linear and angular measurements possible on these fossils are presented in Supplementary Tables 3-21. Throughout, ML = mediolateral; DP= dorsoplantar; PD= proximodistal; MT=metatarsal.

REARFOOT

Talus

<u>U.W. 101-080</u>

U.W. 101-080 is an immature left talus. Its small size and poorly defined borders of the articular surfaces lead to the immature assignation of this bone. There is erosion of the cortex along the entirety of the head, along the distal part of the fibular facet, and proximally along the medial tubercle. The trochlea has damage proximolaterally and distolaterally, but the middle portion of the trochlea is well preserved with cortex that extends to the lateral rim of the fibular facet. The head is eroded dorsally and plantarly, but there is cortex medially and laterally allowing for a head width measure. Plantarly, there is damage both proximally and distally along the rim of the proximal calcaneal facet. The head exhibits mild torsion. The neck is long. The trochlea is flat ML with only a subtle hint of a midline groove. Damage to the lateral fibular facet exposes vertically oriented trabecular bone. The total height from the trochlear rim to the proximal calcaneal facet (midline of fibular facet) is 13.4 mm DP. The total length of the fibular facet is approximately 17.5 mm. The proximal part of the bone yields little information. There is a small lateral tubercle, but the medial tubercle is not preserved and there is no evidence of the *m. flexor hallucis longus* (FHL) tendon groove. The cotylar fossa is damaged but appears to weakly flare medially. Just inferior to the eroded tibial facet is a small pit for the insertion of the deltoid ligament. The proximal calcaneal facet is mildly concave PD and flat ML. Only 13.0 mm PD remains of what would have been a longer facet but the ML dimension are well-preserved along its length and are a maximum of 11.6 mm. There is a 2.2 mm ML sinus tarsi separating the proximal calcaneal facet from the distal, which is badly eroded. The distal calcaneal facet is teardrop shaped (widening distally) and preserves 15.6 mm of its PD length.

<u>U.W. 101-148/149</u>

U.W. 101-148/149 is a nearly complete adult left talus. It was recovered in two parts that conjoin cleanly and were successfully refit. The lateral side of the bone is quite well preserved. Medially, there is some erosion around the head, exposing underlying trabeculae, and the most proximal parts of both the medial tubercle and the proximal calcaneal facet have been sheared away. The trochlea is well-preserved save for the crack running longitudinally along the medial side. In lateral view, the plantolateral part of the head has some exposed trabeculae. The trochlea is

mildly wedged. There is a weak groove medially positioned along the PD axis of the trochlea dividing it into a wider lateral (11.2 mm ML at midpoint) than medial (8.9 mm ML at midpoint) area. The neck is pocked with several foramina. Relative to the trochlea, the neck and head are medially angled. Laterally, the fibular facet is vertically oriented relative to the talar trochlea and then grades smoothly into the laterally projecting base of the fibular facet. The lateral fibular facet is approximately 25.0 mm PD, and 20.2 mm in DP height. The lateral neck, along the distal border of the lateral trochlea possesses a tubercle for the anterior talofibular ligament**.** In proximal view, the lateral tubercle is preserved, as is the approximately 5 mm ML wide groove for the FHL tendon, which descends obliquely dorsolaterally to plantomedially. Medially, the cotylar fossa is mildly cupped and flares 3.6 mm from the trochlear body. The medial facet is 23mm PD in length, and a maximum of 8.0 mm DP at the cotylar fossa and becomes narrow proximally. Plantar to the medial facet is a pit for the insertion of the deltoid ligament. The head is well-preserved, and exhibits considerable torsion. Inferiorly, the proximal calcaneal facet is well-preserved. It is strongly concave PD and relatively flat ML. It is 27.6 mm PD, and a maximum of 17.8 mm ML, though the facet narrows proximally. Relative to the rims of the facet, the deepest point is 5.7 mm. Medial to the facet is a small distal calcaneal facet. A sinus tarsi 3.0 mm wide separates the two facets. The distal facet is PD concave proximally, but flattens distally. It is 17.4 mm PD and 10.6 mm ML. Between the distal calcaneal facet and the head is a small impression for the calcaneonavicular (spring) ligament.

U.W. 101-520

U.W. 101-520 is a complete, but quite damaged, adult left talus. There is severe surface erosion of the cortex along the dorsal border of the head, the plantar part of the head, the proximolateral fibular facet, and the medial and lateral tubercles. Most severe is the damage to the medial and proximal trochlea, where trabecular bone is exposed. Trochlear wedging is moderate. Distal to the trochlea is a relatively long neck. The talar head is preserved well enough to measure a reliable head width, but the DP height of 12.8 mm is a minimum due to damage both plantarly and dorsally. The head exhibits torsion. The fibular facet flares laterally. It is about 21.1 mm PD and 18.8 mm DP. The lateral trochlear neck junction is damaged, eliminating any evidence of an anterior talofibular ligament tubercle. The cotylar fossa is only mildly cupped, and flares medially 2.9 mm. Just plantar and proximal to the medial facet is a small pit for the insertion of the deltoid ligament. In proximal view, the trochlea appears quite flat mediolaterally, though damage medially prevents characterization of midline keeling. The groove for FHL is present and best preserved plantarly, where it is 4.5 mm wide. Plantarly, there is a relatively well-preserved proximal calcaneal facet that is strongly convex PD and flat ML. It is 25.4 mm PD, 14.8 mm ML and 4.9 mm DP deep. A well vascularized sinus tarsi is medial to this facet and is about 2 mm wide. Medial to the sinus tarsi is a damaged distal calcaneal facet. Only 17.5 mm PD and 9.4 mm ML of the cortex is preserved. It is concave proximally and flattens distally. It is flat ML along the length of the facet.

U.W. 101-910

U.W. 101-910 includes an immature left talus and associated navicular. It is small and lacks the defined borders found in adult tali. The head is well-preserved save for some surface erosion medially and plantarly. There is some additional surface erosion at the distal junction between the cotylar fossa and the talar neck. The lateral part of the bone has been sheared away, exposing trabeculae. There is some damage proximoplantarly, removing any evidence for the medial and

lateral tubercles. Plantarly, there is erosion around the proximal and distal rim of the proximal calcaneal facet and laterally along the distal calcaneal facet. The trochlea has a weak but palpable central groove separating the medial and lateral articular surfaces. The trochlea curves distally and terminates in a sulcus that is pocked with many vascular foramina. The neck is a quite long. Despite some erosion around the edges, the head is preserved well enough to accurately measure the ML width and DP height. There is considerable head/neck torsion. The cotylar fossa very weakly extends medially. The medial facet is 16.8 mm PD and it is a maximum of 6.2 mm DP high, which tapers proximally. Plantar to the medial facet is a well-developed pit for insertion of the deltoid ligament. The proximal calcaneal facet is mildly concave PD and flat ML and preserves only 13.0 mm PD and 10.7 mm ML of what would have been a larger facet. The distal calcaneal facet is teardrop shaped and is concave PD (mostly proximally), flattens distally and is flat ML. It is 16.2 mm PD and 12.2 mm ML, which are complete measures. The two calcaneal facets are separated by a sinus tarsi that is 2.7 mm wide. There is a small patch of cortical bone between the navicular facet and the distal calcaneal facet that is flat and serves as the impression of the calcaneonavicular (spring) ligament.

U.W. 101-1031

U.W. 101-1031 is a fragmentary adult left talus that preserves the distal half including the head, distal trochlea and part of the fibular facet. Preserved are a minimum of 32.8 mm PD, 32.9 mm ML, and 21.6 mm DP of the bone. It was recovered in several pieces that conjoin along the neck, connecting the head to the trochlea. The plantar part of the bone has been sheared away and little remains of the medial or proximal parts. There are three very small trabecular filled fragments in association that cannot be reconnected to the main fossil. The head is damaged dorsomedially and plantarly and preserves only 12.9 mm DP. The head and neck exhibit torsion. The neck is long. Plantarly, the bone is almost entirely sheared away save for a very small portion (12.2 mm PD x 6.6 mm ML) of the proximal calcaneal facet. The trochlea is flat ML. The fibular facet is preserved and flares laterally.

U.W. 101-1215

U.W. 101-1215 is a fragmentary left talus preserving part of the lateral body, trochlea and fibular facet. Medially, the body is sheared, exposing trabeculae and a very thin cortical shell. Laterally, the cortex is sheared away proximally, exposing trabeculae. The preserved fibular facet is about 15 mm high DP, which is close to the true measure. It is 20.9 mm PD from the distal edge with the talar neck to the proximal edge of the lateral trochlear body. The fibular facet flares only 3.3 mm ML, though it is eroded and this is a minimum. The trochlear body is flat ML. Distally, a small portion of the body/neck junction is preserved and a small tubercle can be detected for the anterior talofibular ligament. Plantarly, the proximal calcaneal facet preserves only a 12.6 mm ML and 12.3 mm PD patch of cortex which is mildly convex both ML and PD.

U.W. 101-1417

U.W. 101-1417 is a small, nearly complete adult right talus. The only significant damage is to the most proximomedial corner of the bone, shearing away the medial tubercle and the proximal part of both calcaneal facets. The tip of the fibular facet is also mildly damaged. There is some surface erosion around the medial rim and less so around the lateral rim of the talar head. The trochlea is mildly wedged. There is a weak groove medially positioned along the PD axis of the trochlea dividing it into a wider lateral (10.4 mm ML at midpoint) than medial (8.1 mm ML at

midpoint) area. Distal to the trochlea is a long neck separating the trochlear body from the head. Both the medial and lateral parts of the trochlea extend distally, creating a small U-shaped pocket at the body neck junction, which is pocked with several foramina. Relative to the trochlea, the neck and head are medially angled. Laterally, the fibular facet is vertically oriented relative to the talar trochlea and then grades smoothly into a laterally projecting fibular facet. The lateral fibular facet is approximately 20.5 mm PD, and 14.2 mm in DP height. The lateral neck, along the distal border of the lateral trochlea is rugose, but an obvious tubercle for the anterior talofibular ligament cannot be palpated. In proximal view, the lateral tubercle is preserved, as is a very small portion of the most dorsal part of the groove for the FHL. Medially, the cotylar fossa is mildly cupped and flares 5.6 mm medially from the trochlear body. The distal rim of the cotylar fossa is well-developed and has a dorsally projecting lip. The medial facet is 19.9 mm PD in length, and a maximum of 8.6 mm DP at the cotylar fossa, becoming narrower proximally. Plantar to the medial facet is a pit for the insertion of the deltoid ligament. The head is well-preserved and exhibits torsion. Inferiorly, the proximal calcaneal facet is strongly concave PD and relatively flat ML. It is 22.5 mm PD, which is a minimum due to erosion along both the proximal and distal edges of the bone, and a maximum of 14.8 mm ML. Relative to the rims of the facet, the deepest point is 4.1 mm, though this is a minimum because the rims are eroded. Medial to the facet is a small distal calcaneal facet that is damaged both proximally and distally, and thus preserves only 10.3 mm PD and 9.3 mm ML of what would have been a larger facet. A 3.2 mm wide sinus tarsi separates the two facets. Between the distal calcaneal facet and the head is a small impression for the calcaneonavicular (spring) ligament.

U.W. 101-1623:

U.W. 101-1623 is a fragmentary immature right talus. It is quite small and was found in association with other immature pedal elements. The specimen preserves most of the head and talar neck and part of the distal trochlea. The head is abraded plantarly. The neck is well preserved dorsomedially but eroded along other surfaces. The distal talar trochlea is preserved; what remains of the rest of the trochlea is heavily eroded and consists of exposed trabecular bone. The cotylar fossa is weakly cupped and extends 1.8 mm medially; it is 7.8 mm DP. Inferior to the medial cotylar fossa is a very well-developed pit for the deltoid ligament. Plantarly the sinus tarsi is well-preserved though only a small area of posterior calcaneal articular surface remains and this is concave.

Calcaneus

U.W. 101-724

U.W. 101-724 is a fragmentary adult left calcaneus. The entire plantar and proximal surfaces are sheared away. Both the lateral and medial parts of the bone are minimally preserved. Distally, the cuboid facet is well-preserved except for some erosion plantarly and dorsally. Dorsally, both talar facets are reasonably well-preserved, though the proximal talar facet is damaged proximally and the distal talar facet is damaged medially. The sustentaculum tali is damaged medially, exposing underlying trabecular bone. The proximal talar facet is flat ML and convex PD, though it becomes more PD curved proximally. The distal talar facet preserves only a small piece (9.6 mm PD; 4.9 mm ML) which is concave PD and flat ML. The sulcus calcanei separates the two facets and is 3.2 mm wide. Running under the sustentaculum tali is a prominent groove for the FHL tendon. Distally, the cuboid facet is concave DP and mildly convex ML. The bone appears

to have a subtle dorsal rim which is mildly curved, unlike the flat shape in U.W. 101-907. The facet for the cuboid beak extends medially and proximally into a concave region. There is a ridge of bone separating the distal cuboid facet, and the concavity for the beak which is pitted with several holes. In dorsal view, the preserved lateral edge of the calcaneus forms a right angle with the cuboid facet.

U.W. 101-907

U.W. 101-907 is an immature left calcaneus preserving the entire medial length from the cuboid facet to the metaphyseal surface proximally. The entire lateral side of the bone has been sheared away exposing underlying trabeculae. There is erosion plantomedially, along the medial tip of the sustentaculum tali, along the dorsal body, and minor exfoliation of cortical bone in the cuboid facet. The tuber is almost entirely gone save for the most medial part of the metaphyseal surface, which preserves just a small patch of bone (14.9 mm DP; 7.8 mm ML). There is a crack that runs DP through the bone, centered through the proximal talar facet. Plantarly, the bone is heavily damaged along the lateral edge and proximally. Only small patches of cortex can be seen along the middle and distal part of the plantar calcaneus. The distance from the middle of the proximal talar facet to the metaphyseal surface of the proximal tuber is 26.9 mm, and to the cuboid facet is 20.8 mm. In lateral view, the cuboid facet is plantarly angled relative to the long axis of the bone. The minimum DP height of the distal calcaneus from the sulcus calcanei to the plantar base is 15.5 mm. The cuboid facet flares dorsally and is mildly concave DP and flat ML. The dorsal rim is ML flat and exhibits mild distal extension, especially medially. Medially, the facet extends proximally, onto the medial body of the calcaneus, for the beak of the cuboid resulting in a medially angled cuboid facet. In medial view, the plantar surface is flat, and lacks any beaking of the medial plantar process. A strong groove for the FHL tendon runs under the sustentaculum tali. The sustentaculum tali is proximodorsally to distoplantarly angled relative to the long axis of the calcaneus. Just plantar to the sustentaculum tali are several vascular foramina. Along the medial side of the calcaneal body, just proximal to the proximal talar facet is a small pit. Plantodistally, there is a detectable anterior tubercle for the short plantar ligament. Dorsally, the talar facets are relatively well-preserved. The proximal talar facet is sheared laterally and preserves only 12.9 mm ML, but is complete PD. It is flat ML and mildly convex PD. The distal talar facet is mildly concave PD and flat ML, and grades smoothly into the most distal part of the calcaneus. The two talar facets are separated by an approximately 4mm wide sulcus calcanei.

U.W. 101-1322

U.W. 101-1322 is an adult right calcaneus preserving the entire length of the bone from the cuboid facet to a small piece of the likely Achilles insertion on the proximal tuber. There is heavy damage to the tuber itself with exposed trabeculae around the rim and a large portion of the plantar tuber eroded away. There is moderate erosion along the lateral rim of the proximal talar facet, along the medial rim of the sustentaculum tali, and along the border of the cuboid facet. The distance from the middle of the proximal talar facet to the metaphyseal surface of the proximal tuber is 34.5 mm, and to the cuboid facet is 22.5 mm. In lateral view, the cuboid facet is plantarly angled relative to the long axis of the bone. The maximum DP height of the distal calcaneus from the sulcus calcanei to the plantar base is 15.6 mm. The height from most distal plantar region to the most distal edge of the distal talar facet is 17.7 mm DP, as the cuboid facet flares a bit dorsally. The peroneal trochlea is very weakly developed and is positioned distally, at

the base of the proximal talar facet. There are palpable grooves both dorsally (for *m. peroneus brevis*) and plantarly (for *m. peroneus longus*), with the more plantar groove more pronounced. Extending proximally and plantarly is a strongly developed retrotrochlear eminence. While the lateral plantar process is not preserved, it is clear from the anatomy of the retrotrochlear eminence and what is preserved of the plantar tubercles that the lateral plantar process (or tubercle) would be in a modern humanlike plantar position. Distally, the cuboid facet is mildly concave DP and flat ML. The dorsal rim is ML flat and there is a strong distal extension of the dorsomedial rim as is found in modern human calcanei. There is also an anterolateral corner. Medially, the cuboid facet extends proximally, onto the medial body of the calcaneus, for the beak of the cuboid, resulting in a medial angulation of the entire cuboid facet. In medial view, the plantar surface is flat. There is damage to the medial plantar tubercle, preventing any characterization of medial plantar process beaking. A strong groove for the tendon of FHL runs under the sustentaculum tali. The sustentaculum tali is angled proximodorsally to distoplantarly relative to the long axis of the calcaneus. Just proximal to the proximal and medial talar facet is a small pit. Plantarly and distally, there is a detectable anterior tubercle for the short plantar ligament. The middle portion of the plantar surface is roughened, possibly for the origin of the long plantar ligament. Dorsally, there are two relatively well-preserved facets for the talus. The proximal talar facet is flat ML and mildly convex PD, with a humanlike subtended angle of 89°. The distal talar facet is concave ML and flat PD and blends into the most distal part of the calcaneus. The two facets are separated by a 4.2 mm wide sulcus calcanei. Proximally, the tuber preserves a very small patch of cortex 11.9 mm DP and 10.7 mm ML. The superior groove demarcating the separation between the retrocalcaneal bursa and the Achilles insertion can be palpated. While the remainder of the tuber is eroded away, the general shape of it can be characterized as pear-shaped, with the maximum ML dimensions located plantarly as found in human calcanei.

U.W. 101-1662

U.W. 101-1662 is an immature partial right calcaneus, preserving a small piece of the subtalar joint. It is very small and was recovered in association with other immature pedal elements. The calcaneal body is sheared off completely. The proximal talar facet is well-preserved, moderately convex PD and slightly concave ML. The sulcus calcanei is minimally developed. Distally, some of the cuboid facet is preserved. It is moderately concave ML and possesses a slight dorsomedial rim (as occurs in humans).

MIDFOOT

Navicular

U.W. 101-623

U.W. 101-623 is a poorly preserved right navicular. Only 20.2 mm ML and 17.9 mm DP of the fossil remain. The navicular tuberosity is sheared away and there is heavy erosion around the entire perimeter of the bone. Distally, the facets for the cuneiforms are present but heavily eroded. The talar facet is heavily eroded as well; what is preserved (14.8 mm ML and 12.9 mm DP) is concave both ML and DP. The medial cuneiform facet is flat laterally and slightly ML

convex medially. The intermediate cuneiform facet is flat. The lateral cuneiform facet is weakly concave DP and strongly concave ML.

U.W. 101-811
U.W. 101-811 is a left navicular preserving only some of the talar facet, lateral cuneiform facet, and intermediate cuneiform facet. Only 17.7 mm ML and 13.5 mm DP of the fossil remain. The navicular tuberosity has been sheared away and the medial cuneiform facet is crushed. The plantar part of the bone is badly damaged as well. There is erosion mostly medially stripping away the cortical bone. The talar facet is heavily eroded and what is preserved (11.7 mm ML and 9.0 mm DP) is concave both ML and DP. The intermediate cuneiform facet is flat. The lateral cuneiform facet is concave.

U.W. 101-910
U.W. 101-910 is an immature navicular collected with the immature talus (also U.W. 101-910) and calcaneus (U.W. 101-907). The articular facets for the talus and cuneiforms are reasonably well-preserved, though there is erosion around the rims of all facets making measurements impossible. There is very little cortical bone remaining along the medial, lateral, dorsal, or plantar surfaces and trabeculae are exposed throughout. The talar facet is concave both ML and DP. The medial cuneiform facet is flat plantarly and becomes mildly convex dorsally. The intermediate cuneiform facet is slightly concave ML and very little of the lateral cuneiform facet is preserved. The tuberosity projects medially about 3.4 mm from the edge of the talar facet.

U.W. 101-997
U.W. 101-997 is a small, possibly immature right navicular. The dorsal, plantar, and lateral rims of the specimen are slightly abraded, exposing trabecular bone. The facet for the lateral cuneiform is preserved; the intermediate cuneiform facet is well-preserved along its dorsal but not plantar aspect. The facet for the medial cuneiform is not preserved. Proximally, the talar surface is well-preserved and concave both ML and DP. The base of the tuberosity is preserved but the distal aspect is not.

U.W. 101-1030
U.W. 101-1030 is a left navicular, preserving only 20.6 mm ML and 17.0 mm DP. Only part of the talar facet is preserved, along with parts of all three cuneiform facets. The tuberosity has been sheared away and there is surface abrasion along the dorsal and medial rims. The facet for the talar head is damaged medially and therefore preserves only 16.2 mm of what would have been a slightly wider facet. It is concave ML and DP. Plantomedially, there is a projecting tubercle for the plantar cuneonavicular ligament. The lateral part of the medial cuneiform facet is preserved and is relatively flat. The intermediate cuneiform facet is flat. The lateral cuneiform facet is gently concave and is eroded around the rims and preserves only 6.4 mm ML and 6.3 mm DP.

U.W. 101-1562
U.W. 101-1562 is a right adult navicular. There is heavy erosion along the medial edge and the tuberosity is badly damaged. There is pitting and minimal erosion on distal surface. The talar facet is concave ML and DP. All three cuneiform facets are well-preserved. The lateral cuneiform facet is concave ML. The intermediate cuneiform facet is wedge-shaped and flat. The medial cuneiform facet is ML long, medially concave and laterally convex.

Medial Cuneiform

U.W. 101-1039
U.W. 101-1039 is a left medial cuneiform. The navicular facet has been sheared away exposing underlying trabecular bone. The distal facet for the first metatarsal has been damaged leaving only a small patch of cortex plantarly and medially along the middle portion of the facet. Even here, the cortex is cracked. Medially, the plantar and proximal region are damaged. Laterally, the bone is sheared away proximally, and heavily eroded distally. There is exposed trabecular bone dorsally and medially. The distal part of the facet for the intermediate cuneiform is preserved and in dorsal view is angled relative to the long axis of the bone. Medially, there is a grooved surface just proximal to the edge of the first metatarsal facet running proximodorsally to distoplantarly. Distally, the facet for the first metatarsal is reniform, slightly concave DP and slightly convex ML. In distal view, the first metatarsal facet has a slight lateral indentation.

U.W. 101-1062
U.W. 101-1062 is a left medial cuneiform. There is considerable damage to the plantar, distal, and medial surfaces. Distally, the articular facet for the first metatarsal is eroded away, save for a small patch of cortex dorsolaterally. Laterally, there is surface erosion and exposed trabeculae distally and plantarly. Medially, there is erosion along the plantar and distal parts of the bone, but the single "L"-shaped intermediate cuneiform facet is relatively well-preserved. The proximal part of the facet is 5.0 mm PD and becomes more elongated dorsally. Relative to the DP axis of the navicular facet, the facet for the intermediate cuneiform is angled proximomedially to distolaterally. Proximally, the facet for the navicular is teardrop shaped and concave both ML and DP.

U.W. 101-1535
U.W. 101-1535 is a complete left medial cuneiform associated with the U.W. 101-1534 intermediate cuneiform. The bone is well preserved except for some minor abrasion laterally along the intermediate cuneiform facet and proximally along the navicular facet. Laterally, there is a small facet for MT2. Proximal to the MT2 facet is a DP concave and PD flat facet for the intermediate cuneiform, which is damaged proximoplantarly. Plantar to the facets is a PD oriented groove plantar to which the bone is either damaged (proximally) or still covered in some matrix (distally). The medial part of the bone is roughened and fenestrated with several foramina. Proximoplantarly there is a well-developed tuberosity for the insertion of *m. peroneus longus*. Dorsal to that is a smooth facet for the *m. tibialis anterior* insertion. Distal to that region, the bone projects medially and is grooved DP along the rim of the MT1 facet. The MT1 facet is well-preserved and slightly canted medially. The facet is moderately convex ML dorsally and is ML flatter plantarly.

Intermediate Cuneiform

U.W. 101-1242
U.W. 101-1242 is a small right intermediate cuneiform. It is complete, though there is surface erosion around much of the bone, most apparent along the proximomedial, dorsodistal, distoplantar, laterodorsal, and plantar parts of the bone. The facet for the medial cuneiform is

preserved only distally. The MT2 facet is damaged both dorsally and plantarly and appears to not be fully formed, suggesting that this bone may come from an immature individual. The dorsal surface is raised proximally for the insertion of dorsal cuneonavicular ligament. The preserved part of the lateral cuneiform facet is flat PD and concave DP and is a minimum of 7.6 mm PD and 5.7 mm DP. The plantar part of the bone narrows. Proximally, the navicular facet is flat ML and concave PD. It is triangular in shape, with a straight medial border and a slightly indented lateral border. Dorsally, the MT2 facet is flat both ML and DP, though plantarly, the ML dimension becomes more concave as the medial rim is slightly elevated to articulate with a slightly grooved MT2 base.

U.W. 101-1457

U.W. 101-1457 includes a complete adult right intermediate cuneiform, and is associated with a third metatarsal with the same accession number. There is surface erosion around much of the bone and fading of the edges of the articular surfaces. The dorsal surface is raised proximally for the insertion of the dorsal cuneonavicular ligaments. The distal rim of the dorsal surface is also slightly elevated for the dorsal cuneometatarsal interosseous ligaments. The medial border is angled proximomedially to distolaterally for the articulation with an angled surface of the medial cuneiform. There appears to be a single facet for the medial cuneiform connected dorsally and proximally. Plantarly along the medial edge, there is a well-excavated groove for the plantar intercuneiform ligament. The lateral border preserves a facet for the lateral cuneiform that is flat PD and slightly concave DP. The plantar part of the bone narrows. Proximally, the navicular facet is flat ML and mildly concave PD. It is triangular in shape. The MT2 facet dorsally is flat both ML and DP, though plantarly, the ML dimension becomes more concave to articulate with the grooved MT2 base.

U.W. 101-1534

U.W. 101-1534 is a complete adult left intermediate cuneiform, associated with the medial cuneiform U.W. 101-1535. There is some erosion along the distoplantar surface exposing trabecular bone. The dorsal surface is extensively raised proximally for the insertion of the dorsal cuneonavicular ligament, and exhibits some osteophytic lipping. Medially, there is a single facet for the medial cuneiform that is flat DP, and runs the length of the bone PD. Dorsal to the border with the medial cuneiform there is a region of reactive bone extending dorsally and clearly delineated from the facet for the medial cuneiform. Plantar to the facet, there is some pitting and erosion of the bone. Most prominent in medial view is a proximally hooked osteophytic plantar beak. The lateral surface is well preserved. The facet for the lateral cuneiform is flat PD and concave DP. The edges of the facet are well-developed and somewhat osteophytic plantarly. The plantar part of the bone projects proximally. Proximally, the navicular facet is flat ML and concave PD, becoming strongly concave plantarly towards the beak. It is triangular in shape. The MT2 facet is slightly damaged plantarly. Dorsally, it is flat both ML and DP, though plantarly, the ML dimension is concave to receive the slightly grooved MT2 base.

U.W. 101-1618:

U.W. 101-1618 is a well-preserved left intermediate cuneiform. Slight cortical bone abrasion exposes trabecular bone in spots on the medial surface. Proximally, the navicular facet is abraded along its medial half and the plantar portion is broken off giving this surface a flat rather than concave appearance. Medially, a small portion of the articular surface for the medial

cuneiform is preserved dorsally. Laterally, the surface for the lateral cuneiform is slightly proximoplantarly-dorsodistally canted. The distal articular surface for the MT2 is tall, broad and slightly convex.

U.W. 101-1682:

U.W. 101-1682 is a left intermediate cuneiform. It is small, possibly from an immature individual. A portion of the plantar surface is broken and eroded, exposing trabeculae. There is some erosion and pitting on the dorsal surface. There is minor pitting on the navicular surface and heavy erosion exposing trabeculae on the MT2 surface. Medially, the plantar portion of the medial cuneiform facet is eroded. Laterally, the lateral cuneiform facet is canted proximodorsally to dorsodistally.

U.W. 101-1695:

U.W. 101-1695 is a right intermediate cuneiform. It is well-preserved and complete except for the plantomedial part of the bone, which has sheared away. The dorsal surface is raised proximally for the insertion of dorsal cuneonavicular ligament. The distal rim of the dorsal surface is also slightly elevated, and there is some surface erosion here exposing trabeculae. The medial side is damaged plantarly and there is surface erosion dorsally; the medial cuneiform facet is observable but difficult to characterize. The lateral border preserves a facet for the lateral cuneiform that is flat PD and slightly concave DP. Proximally, the navicular facet is flat ML and mildly concave PD. It is triangular in shape, though the plantar part of the facet has been sheared away. The MT2 facet is damaged plantarly, making the DP depth measurement a minimum of 10.3 mm. Dorsally, it is flat both ML and DP, though the ML dimension becomes more concave plantarly to articulate with a slightly grooved MT2 base.

Lateral Cuneiform

U.W. 101-683

U.W. 101-683 is a left lateral cuneiform. It is complete and well-preserved save for some minor erosion of cortical bone along the rims of the facets. It is quite elongated PD and narrow ML. In dorsal view, the lateral side of the bone is PD longer than the medial, resulting in distomedial angulation of the bone relative to the navicular facet. The dorsal surface is rugose proximally and distomedially. The lateral side of the bone is dominated by a large facet for the cuboid. It is dorsoproximally positioned and moderately concave ML and flat DP. Just proximal to the cuboid facet is a small and slightly angled facet for the MT4 indicating that the metatarsal is slightly recessed into the tarsal row. In lateral view, the bone is PD longer dorsally than plantarly, producing an angulation between the navicular and the MT3 facets. There is a vascularized groove plantar to the cuboid facet leading to a plantarly projecting hamulus which is about 5 mm DP and 4.3 mm ML. In plantar view, the hamulus is separated from the navicular facet by a ML groove (for *m. peroneus longus*) which extends onto the medial side of the bone and terminates about half way to the distal surface..Medially, there is a proximal facet for the intermediate cuneiform, which is slightly eroded distally. There is a grooved surface that separates this proximal facet from the distal one for MT2 which has been eroded away. Distally, there is a large facet for MT3. The facet is straight along the medial edge and angles plantarly. Along the lateral edge of the facet, there is a small indentation that tapers the ML width of the facet plantarly. Proximally, the navicular facet is flat both ML and DP.

U.W. 101-1698:
U.W. 101-1698 is a complete adult right lateral cuneiform. It is well-preserved save for some minor erosion of cortical bone along the rims of the navicular facet and the plantar hamulus. It is PD elongated and ML narrow. In dorsal view, the lateral side of the bone is PD longer than the medial, resulting in a distomedial angulation of the MT3 facet relative to the navicular facet. The dorsal surface is particularly rugose proximally, with a raised proximodorsal rim for the insertion of the cuneonavicular ligament. The lateral side of the bone has a large facet for the cuboid. The facet is dorsoproximally positioned and has some erosion proximally. It is flat DP, and moderately concave ML because of a raised rim plantarly. Just proximally to the cuboid facet is a small facet for the MT4 indicating that the metatarsal is recessed into the tarsal row. In lateral view, the bone is PD longer dorsally than plantarly, producing an angulation between the navicular and the MT3 facet. There is a groove plantar to the cuboid facet leading to a plantarly projecting hamulus which has eroded surfaces both proximally and distally. In plantar view, the hamulus is separated from the navicular facet by a groove for the *m. peroneus longus* tendon. The navicular facet is eroded laterally. It is flat both ML and DP. Medially, there is a proximal facet for the intermediate cuneiform. Distal to the intermediate cuneiform facet, there is a small (5.3 mm PD; 7.5 mm DP) facet for MT2 separated from the intermediate cuneiform facet. Distally, there is a large facet for MT3. The facet has a straight medial border and angles plantarly. Along the lateral edge, there is a small indentation that tapers the ML width of the facet plantarly.

U.W. 101-1734:
U.W. 101-1734 is a small, fragmentary, left lateral cuneiform. The plantar half of the bone is broken off. The lateral and medial sides are heavily eroded with exposed trabeculae. In dorsal view it is PD elongated and ML narrow. Dorsally, the lateral side of the bone is longer PD than the medial, resulting in a distomedial angulation of the facet relative to the navicular facet. In lateral or medial view, the dorsum is PD longer than the plantar surface, resulting in an angulation between the navicular and MT3 facets. The MT3 facet is heavily eroded. The navicular facet is DP concave.

Cuboid

U.W. 101-467
U.W. 101-467 is a fragmentary immature right cuboid. It is heavily damaged, exposing trabeculae around the entirety of the bone except plantarly. In anterior view, the beak projects proximally and is eccentrically oriented. Along the plantar surface, the peroneal groove is preserved, and is 3.2 mm wide and 1.7 mm deep.

U.W. 101-1023
U.W. 101-1023 is a left cuboid preserving much of the proximal and distal surfaces, with minimal preservation of cortical bone on the dorsum. The lateral surface is sheared away and there is surface erosion to the cortex medially, plantarly, and around the rims of the articular facets. The plantar part of the bone is eroded, exposing trabecular bone. Dorsally, the surface is flat. In dorsal view, the beak projects proximally and is eccentrically positioned. Medially, there is an eroded, dorsally positioned facet roughly in the middle portion of the bone for the lateral

cuneiform. In medial view, the beak hooks plantarly. Much of the rest of the medial side of the bone is eroded. Plantarly, there is a small portion (about 5 mm ML) of the peroneal groove present, proximal to which is the cuboid tuberosity. The proximal facet is concave DP and convex ML, and preserves only 12.6 mm DP and 14.6 mm ML of what would have been a larger calcaneal facet. The distal facet preserves only the articulation with MT4, which is a minimum of 12.3 mm DP and 9.3 mm ML. The rims of the facet are eroded away but the surface is flat DP and mildly concave ML.

U.W. 101-1418

U.W. 101-1418 is a well-preserved right cuboid. There is damage laterally, shearing some of the most lateral aspect of the bone and exposing trabeculae. Minor erosion can also be found along the rims of articular facets of this bone. Dorsally, the surface is flat. The proximal and distal rims of the dorsal surface are slightly elevated and rugose. Medially, the bone is well preserved, though some surface erosion has exposed trabeculae around the edges of the bone and the facet for the lateral cuneiform is not well-defined. In medial view, the beak hooks plantarly. The plantar part of the bone is well-preserved. It is dominated by the proximally projecting beak. Distal to the beak is a strongly developed cuboid tuberosity. Along the proximolateral border of the peroneal groove, there is a well-developed os peroneum facet. The groove is well-defined by raised bone both proximally and distally. Medially, the groove smooths and terminates. The proximal facet for the calcaneus is concave DP and convex ML. The distal facet preserves the articulation with MT4 and part of the articulation with MT5, separated by a palpable keel. The MT4 facet is flat ML and DP is moderately concave dorsally and flat plantarly, which would complement a sinusoidal MT4 base. The MT5 facet is slightly convex both ML and DP.

FOREFOOT

First metatarsal

U.W. 101-244

U.W. 101-244 is a complete immature left first metatarsal. There is some damage medially and laterally to the head and the medial base has been sheared away. The shaft is quite well preserved save for minor cracking and flaking of the cortex. The proximal metaphyseal surface is flat dorsally, but dips into a small concave area plantolaterally. Dorsally, the shaft is ML flat and bordered medially and laterally by pillars running the length of the shaft (lateral pillar is more pronounced). Laterally, the area plantar to this thickened pillar of bone is concave and flattens distally. The plantar shaft expands proximally into a deep base. The medial shaft is mildly convex distally, and is concave proximally approaching the base. The dorsum of the metatarsal head is domed and extends above the shaft. The bone texture of the head itself is not smooth, but roughened and undeveloped.

U.W. 101-496a

U.W. 101-496a is a left first metatarsal preserving 47.9 mm from the distal tip of the head to a jagged break in the shaft. The shaft is well-preserved, but there is damage to the cortex around the perimeter of the head, concentrated dorsally, laterally, and plantolaterally. Dorsally, the shaft is ML flat and bordered medially and laterally by pillars running the length of the shaft (lateral pillar is more pronounced). Laterally, the area plantar to this thickened pillar of bone is concave and flattens distally. The medial shaft is mildly convex distally, and becomes concave

proximally near the point of the break. Relative to the flat dorsum of the shaft, the head exhibits very little torsion. The medial cornua and pit for the collateral ligaments are well preserved. There is a well preserved groove between the medial cornua and the plantar base for the *m. flexor hallucis brevis* sesamoid.

U.W. 101-1019

U.W. 101-1019 is a nearly complete left first metatarsal, preserved from the tip of the distal head to the proximomedial part of the base (just proximal to the facet for MT2). There is erosion around the entirety of the base, with only a small patch of cortex revealing the facet for the MT2 preserved plantomedially. The shaft is well preserved with only minor exfoliation of cortex in isolated areas. The head has extensive erosion, with only small patches of cortex preserved plantarly and laterally. The facet for the medial cuneiform preserves 14.9 mm DP and only 9.3 mm ML. It is flat DP and ML and oriented nearly perpendicular to the long axis of the bone. Medially, the rim of the facet curves plantarly, producing a concave rim adjacent to the MT2 facet. A groove separating the dorsal and plantar parts of the facet for the medial cuneiform cannot be detected. Dorsally, the base grades into a flat shaft bordered medially and laterally by thickened cortex (lateral pillar is more pronounced). Laterally, the area plantar to this thickened pillar of bone is concave plantarly and flattens distally. The plantar shaft flattens distally, and merges proximally into a robust base. The medial shaft is mildly convex distally, but quite concave proximally. The facet for MT2 is moderately convex and obliquely angled relative to the shaft. Damage to the base has eliminated the *m. peroneus longus* insertion. In lateral view, the dorsal surface is quite flat, and the plantar surface is concave. Lateral and medial to the eroded head, there are pits for the collateral ligament. Plantarly, there is evidence of medial and lateral cornua, a central keel, between which are grooves for the *m. flexor hallucis brevis* sesamoids.

U.W. 101-1443

U.W. 101-1443 is a complete right first metatarsal. There is minor erosion plantomedially along the base, laterally along the base, and under the lateral cornua of the head. There is some longitudinal cracking, but otherwise the bone is in excellent condition. The facet for the medial cuneiform has some minor erosion plantomedially. It is flat DP and slightly concave ML and oriented nearly perpendicular to the long axis of the bone. Medially, the rim of the facet curves proximally, producing a pronounced rim adjacent to the MT2 contact facet. There is no palpable groove separating the dorsal and plantar parts of the facet for the medial cuneiform. Dorsally, the shaft is flat and bordered medially and laterally by thickened cortex. Laterally, the area plantar to this thickened pillar of bone is concave plantarly and flattens distally. The plantar shaft is rounded, becoming flatter distally, but merging into a robust base proximally. The medial shaft is mildly convex distally, but quite concave proximally. Medially and plantarly, there is a well-developed pit for the insertion of *m. peroneus longus*. Just dorsal to the *m. peroneus longus* insertion is a small contact facet for MT2. In lateral view, the dorsal surface of the shaft is quite flat, and the plantar surface is concave. In distal view, the head is wide dorsally. In lateral view, the head extends dorsally relative to the shaft, producing a domed morphology. Laterally and medially to the dorsum of the head, there are pits for the collateral ligament. Plantarly, there is evidence of medial and lateral cornua, a central keel, between which are grooves for the *m. flexor hallucis brevis* sesamoids. Collected with this specimen is one of the sesamoids (U.W. 101-

1553). It is 9.8 mm PD, 6.4 mm ML, and 4.0 mm DP. It is convex plantarly, and concave dorsally, with a smooth facet for contact with the plantar region of the head.

U.W. 101-1530

U.W. 101-1530 is a nearly complete right first metatarsal, preserving the base to the eroded edge of the damaged head. The shaft is well preserved but there is heavy erosion around the perimeter of the base, and the head is heavily eroded, exposing trabecular bone. The facet for the medial cuneiform is flat DP and concave ML and oriented nearly perpendicular to the long axis of the bone. On the dorsal part of the facet, the rims curve proximally, producing a pronounced concavity. There is a detectable groove separating the dorsal and plantar parts of the facet for the medial cuneiform. Dorsally, the shaft is flat and bordered medially and laterally by thickened cortex. Laterally, the area plantar to this thickened pillar of bone is concave plantarly and flattens distally. Dorsomedially, there is a raised thickening of the cortex. The plantar shaft flattens distally, and merges proximally into a robust, albeit damaged, base. The medial shaft is mildly convex distally, but quite concave proximally. In lateral view, the dorsal surface is flat, rising only dorsomedially, and the plantar surface is concave.

Second metatarsal

U.W. 101-459/461

U.W. 101-459/461 is a right second metatarsal preserving 48.3 mm from the proximal base to a break in the shaft just prior to the metatarsal head. The bone was recovered in two roughly equal pieces that conjoined along the proximal third of the shaft. The base is eroded around its perimeter. The MT3 and medial cuneiform facets are poorly preserved. The facet for the intermediate cuneiform is weakly concave both ML and DP, and angled proximolaterally to distomedially. A thickening of cortex beginning medially spirals across the dorsum of the bone to the laterally side of the distal shaft resulting in slight internal torsion of the shaft. In lateral view, the shaft is relatively straight dorsally and moderately curved plantarly. Plantarly, the more distal part of the tubercle is preserved, but the proximal part is sheared away. What is preserved is oriented parallel to the shaft.

U.W. 101-1022

U.W. 101-1022 is a complete left second metatarsal, recovered in four pieces that conjoin cleanly together. Transverse cracks circle the perimeter of the bone where these four pieces join. The base has some damage plantarly. The cortex is abraded medially, dorsally, and plantarly revealing underlying trabecular bone. There is erosion around the perimeter of the metatarsal head, preventing any head size estimates. However, there remains cortex at the far distal end of the head, allowing for an accurate length measurement. The base is angled proximolaterally to distomedially relative to the long axis of the shaft. The triangular articular surface for the intermediate cuneiform is mildly concave ML, and flat DP. Laterally, there are facets for the MT3 and for the lateral cuneiform. The MT3 facet is dorsolaterally positioned. Proximal to the MT3 facet is a small, abraded facet for the lateral cuneiform. Just plantar to the facet for MT3 is a large pit for the interosseous ligaments. Medially, there is erosion along the facet for the medial cuneiform and the plantar region has been sheared away. Plantar to the medial cuneiform facet is a small indentation for the insertion of the Lisfranc ligament. Still plantar, just distal to the eroded edge is a bulbous convexity that contacts the associated MT1 (U.W. 101-1019). In lateral

view, the shaft is only moderately convex dorsally and concave plantarly. There is subtle thickening of the bone both medially and laterally along the length of the shaft. Damage to the dorsum of the head precludes any characterization of dorsal doming of the metatarsal head. At the shaft-head junction, there is a small tubercle preserved laterally.

U.W. 101-1458

U.W. 101-1458 is a complete right second metatarsal. There is surface erosion plantarly along the base, and along the plantar and lateral aspect of the head. Medially, there is erosion along the facet for the medial cuneiform. The base is angled proximolaterally to distomedially relative to the long axis of the shaft. The triangular articular surface for the intermediate cuneiform is mildly concave ML, and flat DP. Laterally, there are facets for the MT3 and for the lateral cuneiform, clearly delineated from the MT3 facet. Proximal to the MT3 facet, is a small facet for the lateral cuneiform. Just plantar to the facet for MT3 is a large pit for the interosseous ligaments. Plantarly, just superior to a groove for the insertion of the Lisfranc ligament is a small bulbous articular surface that contacts MT1 (U.W. 101-1443). In lateral view, the shaft is straight dorsally and more concave plantarly. There is subtle thickening of the shaft both medially and laterally along its length. The head is slightly domed above the level of the dorsal shaft. Proximal to the head is a small sulcus bordered at the shaft-head junction medially and laterally by small tubercles.

U.W. 101-1499

U.W. 101-1499 is a small, immature right second metatarsal, preserving 30.4 mm from the base to a break in the shaft. There is heavy surface erosion along the shaft and both the dorsal and plantar surfaces of the base. The facet for the intermediate cuneiform is eroded both dorsally and plantarly. Medially, there is erosion along the facet for the medial cuneiform and its dimensions cannot be assessed with accuracy. The base is angled proximolaterally to distomedially. The intermediate cuneiform facet is flat DP and mildly concave ML owing to a proximomedial projection. Laterally, there are facets for MT3 and for the lateral cuneiform. Proximal to the MT3 facet, is a small facet for the lateral cuneiform, though the edges of this facet are not clear. Just plantar to the facet for MT3 is a large pit for the interosseous ligaments. Plantarly and medially, there is a smooth region that contacts the associated MT1 epiphysis (also U.W. 101-1499).

Third metatarsal

U.W. 101-552

U.W. 101-552 is a partial left third metatarsal, preserving 51.9 mm of the base to a break in the shaft just proximal to the head. There is some flaking of cortical bone from the dorsolateral base exposing trabecular bone. Medially, there is considerable damage to the dorsal MT2 facet exposing trabeculae. Plantarly, the large plantar tuber is broken proximally. The facet for the lateral cuneiform has erosion around the rims, and is mildly concave ML and flat DP. In dorsal view, the facet for the lateral cuneiform is strongly angled proximolaterally to distomedially. Dorsally, the shaft is flat proximally and becomes more ML convex distally. Laterally, there is a large facet for MT4 that spills onto the dorsal surface as happens in human MT3s because of the high transverse arch. The facet is mildly concave PD and mildly convex DP. Plantarly, the preserved tuber projects plantarly and is angled proximolaterally to distomedially.

U.W. 101-1035

U.W. 101-1035 is a left third metatarsal, preserving 42.1 mm from a distal break in the shaft to a very small preserved patch of the proximal base. The bone was recovered in two pieces that were refit cleanly. There is heavy damage to the base (mostly plantar), removing much of the anatomy. There is erosion to the proximal dorsal surface of the shaft exposing underlying trabeculae. A fragment of the distal shaft is broken away. The base is angled proximomedially to distolaterally. Despite damage to the base, the narrow, triangular shape of the lateral cuneiform facet is still detectable, preserving 4.5 mm DP and 2.8 mm ML. It appears relatively flat along the medial border, but there is an indentation laterally. Dorsally, the shaft is flat proximally and becomes slightly convex (ML) distally. Medially, there is a dorsally positioned, eroded facet for MT2. In this view, the shaft is moderately curved plantarly and relatively flat dorsally. In plantar view, the projecting plantar tuberosity angles slightly proximomedially.

U.W. 101-1457

U.W. 101-1457 includes a complete and well-preserved third metatarsal, collected with an intermediate cuneiform with the same accession number. A portion of the plantar surface of the head is damaged. The head was collected separately from the shaft was cleanly refit. The facet for the lateral cuneiform has some erosion around the lateral rim. Medially, the MT2 facet is eroded, exposing trabeculae. The lateral cuneiform facet is flat ML and DP. In dorsal view, this facet is angled proximolaterally to distomedially**.** Dorsally, the shaft is flat proximally and becomes more convex distally. The dorsal base is rugose with tuberosities both on the lateral and medial aspects. Medioplantarly to the MT2 facet, there is a proximal sulcus and a large tubercle distally. Plantarly, the base is quite rugose, with a large tubercle for plantar ligaments. Laterally, there is a large facet for MT4, which is mildly concave PD and flat DP. Laterally at the midshaft is a large foramen. Because of damage to the head, the ML width measurement was not possible. Dorsally, there is a small sulcus proximal to the head, and the head is domed, rising above the long axis of the shaft. Medially and laterally, the head is bordered by two tubercles dorsally. In distal view, the head exhibits external torsion relative to the shaft.

U.W. 101-1500

U.W. 101-1500 is a small, immature right third metatarsal, preserving 30.0 mm from the base to a break in the shaft. The dorsal surface is entirely sheared away, preserving only the plantar half of the bone, which is well-preserved. Medially, most of the facet for MT4 has been sheared away. The articular surface for the lateral cuneiform is flat DP and mildly concave ML. Medially, there is a dorsally positioned facet for MT2. Plantar to the facet is a flat and roughened region. Plantarly, the bone tapers to a strong projection.

Fourth metatarsal

U.W. 101-248

U.W. 101-248 is a small, immature left fourth metatarsal preserving 35.9 mm from the base to a break in the shaft. There is cortical bone that has flaked away from the proximal dorsal surface. Medially, the facet for MT3 is badly damaged, and trabeculae are exposed plantarly. The plantolateral part of the bone is well-preserved. The base is damaged plantarly preserving only 4.0 mm DP and 5.4 mm ML of the cuboid facet. What is preserved is flat both ML and DP, and

angled proximolaterally to distomedially. The facet for MT5 preserves a small patch of cortex but is broken plantarly. The lateral part of the base narrows and indents relative to the plantar tuber. The plantar aspect of the proximal base is 'pinched'. The bone twists in a manner which results in noticeable external torsion of the distal shaft.

U.W. 101-269

U.W. 101-269 is a complete right fourth metatarsal, recovered in three pieces that cleanly refit. The base and shaft are very well-preserved save for some minor exfoliation of cortex. The head is damaged plantarly, exposing trabecular bone. The base is angled proximomedially to distolaterally relative to the shaft. The base is robust. The articular facet for the cuboid is very mildly concave ML and mildly convex DP. The MT5 facet is bordered plantarly and distally by a strong groove distal and plantar to which is a large tuberosity for the second *m. plantar interosseous*. Medially, there is a large, flat facet for MT3 which grades weakly into a contact for the lateral cuneiform. A plantar tubercle is large and projecting. Just distal to the base and continuing through midshaft, the bone exhibits considerable external torsion. The head is slightly domed relative to the shaft and there is a small sulcus just proximal to the head. In lateral view, the dorsum of the shaft is quite straight, whereas the plantar region is mildly concave.

U.W. 101-1368

U.W. 101-1368 is an immature right fourth metatarsal, preserving 26.1 mm from the base to a break in the shaft. It is from a slightly larger individual than U.W. 101-248 and is unlikely to be an antimere. There is damage to the proximal dorsal surface, exposing underlying trabeculae. Exposed trabecular bone rings the entirety of the base. Cortical bone has flaked away from the dorsum of the broken shaft. Medially, the facet for MT3 and lateral cuneiform is damaged dorsally, but is preserved otherwise. The contact between the MT3 facet and the lateral cuneiform is difficult to discern as the two blend cleanly into one another. Laterally, there is a small preserved area of contact for MT5. The lateral part of the base narrows and indents relative to the plantar tuber. Only 8.5 mm DP and 6.1 mm ML of the cuboid facet is preserved and is flat DP and slightly concave ML. The base is angled proximolaterally to distomedially. Relative to the base, the shaft externally twists. At the point of the distal break, the shaft is 4.4 mm ML and 6.1 mm DP.

U.W. 101-1456

U.W. 101-1456 is a complete right fourth metatarsal, with only minor surface abrasion to the lateral head, and around the perimeter of the base. The most plantar part of the base appears to have suffered some minor crushing. Laterally, there is minor erosion around the MT5 facet. The base is angled proximomedially to distolaterally relative to the shaft and is robust. The articular facet for the cuboid is very mildly concave ML and mildly convex DP. Distally along the medial base there is a large tuberosity for the second *m. plantar interosseous* and between this tuberosity and the MT5 facet is a grooved and vascularized region. Dorsomedially, there is a small tubercle for the fourth *m. dorsal interosseous*. Medially, there is a large facet for MT3 which grades weakly into a contact for the lateral cuneiform. It is flat PD and mildly concave DP**.** A plantar tubercle is large and separated from the contact facet for MT3 by a grooved region. The shaft exhibits considerable external torsion. The head is domed relative to the shaft and there is a small sulcus just proximal to the head. The sulcus is angled distomedial to proximolateral. In lateral view, the dorsum of the shaft is quite straight, whereas the plantar region is mildly concave.

There are two tubercles at the head-shaft junction dorsally, and plantarly, the head terminates in two plantar cornua.

Fifth metatarsal

U.W. 101-518
U.W. 101-518 is a partial right fifth metatarsal preserving 45.6 mm of the base and shaft. The shaft is well-preserved with only minor cortical exfoliation in places. There is plantar damage to the tuberosity exposing trabecular bone. In dorsal or plantar view, the distal shaft bends laterally and is concave. Proximally, the base flares. The dorsal base possesses a medial groove and laterally is dominated by the tuberosity of the metatarsal. Medially, there is a large tubercle for the origin of *m. flexor digiti minimi*. The facet for MT4 is flat. The cuboid facet is flat ML and mildly concave DP, and angled proximolateral to distomedial. At the break in the shaft, the bone is DP compressed: 8.4 mm ML and 5.3 mm DP, dimensions that remain consistent near approximate midshaft as well.

U.W. 101-1412
U.W. 101-1412 is a partial right fifth metatarsal preserving 37.9 mm of the shaft and the distal part of the MT4 facet. The head and base are not preserved. The shaft is well-preserved with only minor cracks along the long axis of the bone. At the point of the distal break, the shaft is DP compressed: 6.6 mm ML, 5.2 mm DP. The facet for MT4 is flat.

U.W. 101-1439
U.W. 101-1439 is a complete right fifth metatarsal. It is well-preserved except for some minor damage along the plantar aspect of the tuberosity, the plantolateral part of the head, and the dorsal aspect of the MT4 facet. The shaft is well-preserved with only minor longitudinal cracking. In dorsal or plantar view, the distal shaft bends laterally. The dorsal base possesses a medial groove parallel with the cuboid facet and bordered distally by raised rugose bone. Laterally, the dorsum is dominated by the tuberosity of the metatarsal. Medially, there is a large tubercle for the origin of *m. flexor digiti minimi*. Just lateral to the tuberosity is a well-developed groove for the *m. abductor digiti minimi* tendon. The facet for MT4 is flat PD. The cuboid facet is angled proximolateral to distomedial and is flat both ML and DP.

Metatarsal fragments

U.W. 101-497
U.W. 101-497 is an unsided metatarsal shaft 19.0 mm PD. The cortical bone exhibits some flaking but is in reasonably good condition. It is expanded DP distally. Proximally the diaphysis is more circular but still DP ovoid.

U.W. 101-750:
U.W. 101-750 is an unsided metatarsal shaft fragment preserving the proximal portion of the diaphysis. Neither articular end is preserved.

U.W. 101-801:

U.W. 101-801 is an unsided metatarsal head fragment . The metatarsophalangeal joint surface is well-preserved and grades dorsally.

U.W. 101-869:
U.W. 101-869 is an unsided metatarsal head fragment. The metatarsophalangeal joint surface is well-preserved and grades dorsally.

U.W. 101-1437:
U.W. 101-1437 consists of two small fragments of metatarsal shaft with minor cortical erosion; no informative anatomy remains.

U.W. 101-1444:
U.W. 101-1444 is a metatarsal shaft fragment 19.5 mm PD. The cortical bone is preserved but heavily scored with longitudinal cracks.

U.W. 101-1499:
U.W. 101-1499 is a right MT1 epiphysis associated with an immature MT2 (also U.W. 101-1499). It is 15.5 mm DP and 11.2 mm ML. The articular facet for the medial cuneiform is flat both DP and ML. The most medial side is slightly pinched in giving the surface a moderately reniform shape. The insertion area for *m. peroneus longus* is bulbous, and does not yet possess the concavity found in adult MT1s. The metaphyseal surface is rough and plantomedially possesses a distally projecting extension.

U.W. 101-1513:
U.W. 101-1513 is a small fragment of metatarsal preserving the diaphyseal midpoint towards the proximal end, 15.8 mm PD.

U.W. 101-1559:
U.W. 101-1559 is a metatarsal shaft fragment 15.5 mm PD.

U.W. 101-1585:
U.W. 101-1585 is a fragmentary, unsided metatarsal 29.0 mm PD, preserving a portion of the proximal shaft near the base. Remaining cortical bone is eroded and embedded with soil sediment.

Proximal pedal phalanges

U.W. 101-082
U.W. 101-082 is a left hallucial proximal phalanx. There is a large piece of the proximal, dorsal, and medial part of the bone missing, and there is erosion around the head and base. This bone was refit near the midpoint of the diaphysis. The lateral half of the metatarsophalangeal joint surface is preserved, presenting ML concavity and DP flatness. The head is reasonably well-preserved; the medial portion is eroded exposing trabecular bone. The head is gently keeled.

U.W. 101-504

U.W. 101-504 is a left proximal pedal phalanx. There is damage to the lateral part of the base and some surface erosion along the dorsum of the head and along the medial rim of the base. The base is dorsally canted. The head is quite keeled.

U.W. 101-725
U.W. 101-725 is a proximal pedal phalanx preserving much of the shaft and head. The base is heavily eroded. The head has some erosion along the dorsum and is quite keeled.

U.W. 101-976
U.W. 101-976 is a proximal pedal phalanx. The head is badly damaged. The base is damaged plantarly and along the medial and lateral rims.

U.W. 101-1013
U.W. 101-1013 is a left proximal pedal phalanx with a bony callosity present on the proximal aspect of the diaphysis that is a healed fracture, confirmed upon radiographic examination. Damage around the plantar, medial, and lateral rims of the base preclude a ML base measure. The base is dorsally canted. The midshaft is circular. Plantarly, the cornua extends more proximally on the lateral side.

U.W. 101-1024
U.W. 101-1024 is the proximal 14.3 mm of a left hallucial proximal phalanx. The plantar part of the bone has been sheared away and most of the shaft and the entirety of the head were not recovered.

U.W. 101-1034
U.W. 101-1034 is a left proximal pedal phalanx. There is damage around the base precluding any measurements, though there is a small patch of cortex still preserved allowing for a length measure. The head is weakly keeled.

U.W. 101-1148
U.W. 101-1148 is a well-preserved proximal pedal phalanx. The base is robust plantarly and is dorsally canted. The head is quite keeled.

U.W. 101-1395
U.W. 101-1395 is a well-preserved proximal pedal phalanx. There is slight abrasion of the cortical bone around the rim, exposing very small patches of trabecular bone intermittently. There is a callous ringing the dorsal, medial, and lateral surfaces where the angle occurs. The diaphysis is slightly displaced; this displacement is confirmed upon radiographic imaging. The base is dorsally canted. The head is quite keeled. A second, smaller, more damaged proximal pedal phalanx was found in association. It preserves only 15.7 mm of the shaft and eroded base.

U.W. 101-1419
U.W. 101-1419 is an almost complete proximal hallucial phalanx. There is considerable damage to the medial and lateral rims of the base, and the head has eroded away leaving 23.3 mm of the base and shaft. Plantarly, the proximal portion of the diaphysis is slightly eroded.

U.W. 101-1441
U.W. 101-1441 is a well-preserved proximal pedal phalanx. There is some damage to the dorsal surface of the head and to the dorsal rim of the base. The base is dorsally canted. The head is quite keeled.

U.W. 101-1442
U.W. 101-1442 is an almost complete proximal hallucial phalanx, preserving 22.6 mm of the shaft and base. There is some damage around the dorsal rim of the base, and the head has eroded away. Plantarly, the base is well preserved with the proximal excursion more developed medially. The surface is ML concave, with a small ridge along the dorsal midline proceeding slightly proximally.

U.W. 101-1452
U.W. 101-1452 is a well-preserved proximal hallucial phalanx. There is some damage to the dorsal rim of the base. There is no visible curvature of the shaft. The head is keeled.

U.W. 101-1557:
U.W. 101-1557 is a proximal pedal phalanx. It preserves the distal head and most of the diaphysis. The head is well-preserved, and keeled. There is ML flaring at the distal end. The plantar aspect of the shaft is concave.

U.W. 101-1657
U.W. 101-1657 is a small, immature pedal proximal phalanx preserving the phalangeal shaft and head. The trochlear surface is worn and trabecular bone is exposed.

Intermediate pedal phalanges

U.W. 101-550
U.W. 101-550 is an intermediate pedal phalanx. The head is well-preserved and strongly keeled. Part of the lateral base and shaft is damaged, exposing trabecular bone.

U.W. 101-661
U.W. 101-661 is an intermediate pedal phalanx. There is damage to the dorsal base. The head lacks the keeling found in the other phalanges. Flexor ridges at proximal end of plantar surface are visible.

U.W. 101-988
U.W. 101-988 includes an intermediate pedal phalanx and an associated distal phalanx with the same catalogue number. The head is reasonably well-preserved though its plantar rim from its midline to the lateral aspect is eroded, exposing trabecular bone. It is strongly keeled. The diaphysis is well preserved with minimal pitting and abrasion. The base is vaguely bipartite, and has minor damage plantarly.

U.W. 101-1042
U.W. 101-1042 is an intermediate pedal phalanx. There is some minimal damage plantarly along the base. The head is keeled and the base is bipartite.

U.W. 101-1399
U.W. 101-1399 is an intermediate pedal phalanx. There is very minor damage to the plantar rim of the base. The head is strongly keeled.

U.W. 101-1438
U.W. 101-1438 is an intermediate pedal phalanx. There is very minor circular damage to the plantar rim of the base. The head is strongly keeled.

U.W. 101-1484
U.W. 101-1484 is an immature intermediate pedal phalanx. It was collected with an epiphysis of a hallucial pedal phalanx (U.W. 101-1483). The proximal surface is irregular for the congruent surface of the epiphysis. There is minor damage plantarly to the metaphysis. The head is keeled.

U.W. 101-1549:
U.W. 101-1549 is an intermediate pedal phalanx. It is well-preserved with a pit in the cortical bone on the base located plantarly at the midline. The base is DP concave and ML convex. The head is slightly abraded and mildly keeled.

U.W. 101-1575:
U.W. 101-1575 is an intermediate pedal phalanx fragment preserving the head and distal shaft. The diaphysis is sheared off obliquely and coronally at its proximal end. The head is keeled.

U.W. 101-1587:
U.W. 101-1587 is a complete intermediate pedal phalanx, with minor pitting of cortical surface. There is minor erosion on the plantar edge of the base exposing trabeculae. The lateral side of the base is flared relative to the diaphysis and medial side of base, possibly the result of degeneration due to asymmetrical loading. The head is relatively flat.

U.W. 101-1591:
U.W. 101-1591 is an intermediate pedal phalanx. The distal half of the bone has sheared away. The base is well preserved, though shows a pit plantarly at the midline. This surface is DP concave and ML weakly convex.

U.W. 101-1594:
U.W. 101-1594 is a complete intermediate pedal phalanx. It is presently heavily embedded with soil sediment. Erosion at the base and head exposes trabeculae. There is pitting of the diaphyseal cortical bone.

U.W. 101-1625:
U.W. 101-1625 is an intermediate pedal phalanx. The specimen preserves the distal half of the diaphysis and head. The diaphyseal cortical bone exhibits minor exfoliation around its circumference. There is slight abrasion of the plantar and dorsal aspects of the distal head. The head is mildly keeled.

Distal pedal phalanges

U.W. 101-988
U.W. 101-988 consists of a distal pedal phalanx associated with an intermediate phalanx with the same catalogue number. The base is well preserved dorsally; the plantar aspect of the articular surface is eroded with trabecular bone exposed. The diaphysis shows some abrasion with a bit of trabecular bone exposed dorsally but not plantarly. The plantar aspect of the distal head shows minimal erosion.

U.W. 101-1010
U.W. 101-1010 is a distal pedal phalanx. The distal end flares into a small tuft, which angles dorsally.

U.W. 101-1526:
U.W. 101-1526 is a distal pedal phalanx. The distal end exhibits minor tufting, the diaphysis is concave plantarly and flat dorsally. The base is flat and slightly canted dorsally.

U.W. 101-1550
U.W. 101-1550 is a distal pedal phalanx. It is well preserved; only the dorsal aspect of the head shows minor abrasion exposing cortical bone. The base is slightly DP concave and appears ML flat. The head is tufted.

U.W. 101-1551:
U.W. 101-1551 is a right hallucial distal phalanx. The medial aspect of the base has broken off. The head is broken medially. The plantar head is smooth and angled dorsally. The pit for the flexor hallucis longus tendon is quite deep and pronounced. Laterally, the base possesses a proximally projecting hook.

U.W. 101-1576:
U.W. 101-1576 is a complete distal pedal phalanx. The base is DP elongated at the midline, with reinforced, thick rims. The diaphysis is very short and ML compressed. The head forms a pronounced tuft.

Phalangeal fragments

U.W. 101-884:
U.W. 101-884 is a proximal pedal phalanx fragment preserving the diaphysis and head. The base is broken; there is minor cortical erosion of the diaphysis and head, exposing trabeculae.

U.W. 101-1118:
U.W. 101-1118 is a phalanx diaphyseal fragment 13.8 mm PD; the head and base are missing. There is heavy cortical bone erosion with pitting throughout the diaphysis, which has a ML width of 5.6 mm, and DP height of 4.4mm.

U.W. 101-1483

U.W. 101-1483 is the epiphysis of an immature proximal hallucial phalanx. It is 11.9 mm ML and 10.2 mm DP and the articular surface is concave in both directions. The epiphyseal surface is roughened and maximally 4.9 mm thick DP.

U.W. 101-1589:
U.W. 101-1589 is a pedal phalanx fragment. The base and head are broken off, and there is minor cortical erosion of the diaphysis. The most distal edges of the flexor ridges are barely noticeable. The proximal end is DP and ML expanded relative to the diaphysis.

U.W. 101-1592:
U.W. 101-1592 is a fragmentary pedal phalanx. It is missing its dorsal base, and the plantar base exhibits mild erosion. The diaphysis is flat dorsally and concave plantarly.

U.W. 101-1595:
U.W. 101-1595 is a fragmentary pedal phalanx. The head is broken with the plantar aspect of one side missing. The dorsal aspect of the rim is eroded slightly. The base is ML convex and DP flat. The plantar surface is concave.

UW-101-1598:
U.W. 101-1598 is a fragmentary pedal phalangeal shaft. Posteriorly a medullary cavity is exposed.

Supplementary References

1. DeSilva, J.M. Functional morphology of the ankle and the likelihood of climbing in early hominins. *PNAS*. **106**, 6567-6572 (2009).

2. Harcourt-Smith, W.E.H. *Form and function in the hominoid tarsal skeleton*. Ph.D. Thesis, University of London (2002)

3. Jungers, W. L. et al. The foot of *Homo floresiensis*. *Nature* **459**, 81-84 (2009).

4. Latimer, B. & Lovejoy, C.O. The calcaneus of *Australopithecus afarensis* and its implications for the evolution of bipedality. *Am. J. Phys. Anthropol.*, **78**: 369-86 (1989).

5. Zipfel, B. *et al*. The foot and ankle of *Australopithecus sediba*. *Science* **333**, 1417-1420 (2011).

6. DeSilva, J.M. Revisiting the midtarsal break. *Am. J. Phys. Anthropo.*,**141**,245-258 (2010).

7. DeSilva, J.M. et al. The lower limb and the mechanics of walking in *Australopithecus sediba*. *Science* **340**, 1232999 (2013).

8. Day, M.H. & Wood, B.A. (1968). Functional Affinities of the Olduvai Hominid 8 Talus. *Man*, New Series, **3**(3), 440–455.

9. Schultz, A.H. The relative lengths of the foot skeleton and its main parts in primates. *Symp. Zool. Soc. Lond.* **10**, 199-206 (1963).
